# Psychiatric symptoms are not associated with circulating CRP concentrations after controlling for medical, social, and demographic factors

**DOI:** 10.1038/s41398-022-02049-y

**Published:** 2022-07-12

**Authors:** Leandra K. Figueroa-Hall, Bohan Xu, Rayus Kuplicki, Bart N. Ford, Kaiping Burrows, T. Kent Teague, Sandip Sen, Hung-Wen Yeh, Michael R. Irwin, Jonathan Savitz, Martin P. Paulus

**Affiliations:** 1 Laureate Institute for Brain Research, Tulsa, OK 74136 USA; 2 Department of Computer Science, Tandy School of Computer Science, The University of Tulsa, Tulsa, OK 74104 USA; 3 Department of Pharmacology & Physiology, Oklahoma State University, Center for Health Sciences, Tulsa, OK 74107 USA; 4 Department of Surgery and Department of Psychiatry, University of Oklahoma-School of Community Medicine, Tulsa, OK 74135 USA; 5 Division of Health Services & Outcomes Research, Children’s Mercy Kansas City, Kansas City, MO 64108 USA; 6 Department of Psychiatry and Behavioral Sciences, UCLA Geffen School of Medicine, Los Angeles, CA 90095 USA; 7 Oxley College of Health Sciences, The University of Tulsa, Tulsa, OK 74199 USA

**Keywords:** Molecular neuroscience, Predictive markers

## Abstract

Elevated serum concentrations (>3 mg/L) of the acute-phase protein, C-reactive protein (CRP), is used as a clinical marker of inflammation and is reported to be a strong risk factor for cardiovascular disease. In psychiatric populations, CRP concentration is reported to be higher in depressed versus healthy individuals. Positive associations between CRP and depression have been established in both clinical and community samples, but effect sizes are attenuated after controlling for confounding variables. Similarly, emerging research has begun to draw a link between inflammation, symptoms of anxiety, and substance abuse. Given the high level of comorbid anxiety and substance use disorders in many depressed populations, this study examined whether depression (Patient Health Questionnaire 9 [PHQ-9]) and substance use-related (Drug Abuse Screening Test [DAST]) symptoms were associated with CRP concentrations in the blood after adjusting for relevant medical, social, and demographic covariates in a large sample undergoing screening for several transdiagnostic psychiatric research studies. A total of 1,724 participants were analyzed for association of CRP with variables using multivariate linear regression. An unadjusted model with no covariates showed that PHQ-9 was significantly associated with CRP in All (*β* = 0.125), Female (*β* = 0.091), and Male (*β* = 0.154) participants, but DAST was significantly associated with CRP in males only (*β* = 0.120). For the adjusted model, in both males and females, mood-stabilizer treatment (*β* = 0.630), opioid medication (*β* = 0.360), body mass index (*β* = 0.244), percent body fat (*β* = 0.289), nicotine use (*β* = 0.063), and self-reported sleep disturbance (*β* = 0.061) were significantly associated with increased CRP concentrations. In females, oral contraceptive use (*β* = 0.576), and waist-to-hip ratio (*β* = 0.086), and in males, non-steroidal anti-inflammatory drug use (*β* = 0.367) were also associated with increased CRP concentrations. There was no significant association between CRP and individual depressive, anxiety, or substance use-related symptoms when covariates were included in the regression models. These results suggest that associations between circulating CRP and the severity of psychiatric symptoms are dependent on the type of covariates controlled for in statistical analyses.

## Introduction

C-reactive protein (CRP) is an acute-phase protein synthesized primarily by hepatocytes in the liver and cells in the gall bladder [1]. CRP gene transcription is initiated in response to tissue damage, infection, or proinflammatory cytokines such as interleukin-6 (IL-6) and tumor necrosis factor (TNF) [2, 3]. CRP is used as a clinical marker of inflammation with elevated serum concentrations (>3 mg/L) shown to be a strong independent risk predictor of cardiovascular disease as recommended by the Center for Disease Control and the American Heart Association [4–6]. This concentration threshold (>3 mg/L) has also been associated with increased risk for future episodes of depression, and on average, CRP has been reported to be elevated in depressed versus healthy populations [7–10]. Cross-sectional studies have reported that depressed subjects display elevated levels of inflammatory cytokines, such as IL-6 and TNF, as well as CRP compared to controls [8, 11–13]. Positive associations between CRP and depression have been established in both clinical and community samples, but effect sizes are attenuated after controlling for confounding variables [14–17]. A systematic review by Horn et al. (2018), including 26 studies in the most methodologically rigorous stage, reported a significant but attenuated relationship between circulating CRP concentrations and depression, with a small effect size (*r* = 0.05) after adjusting for confounding variables such as age, sex, obesity, and others [9, 18]. One of the key factors responsible for variability in outcome between studies is the extent to which confounding demographic, psychosocial, lifestyle, and medical variables that influence levels of inflammation are controlled for in statistical analyses [4, 18, 19]. Resolving the extent to which the relationship between CRP and psychiatric symptoms is moderated by psychosocial and health factors is important not only because it may inform our understanding of the pathophysiology of depression, but because it may provide opportunities for intervention [9].

This study aimed to examine whether self-reported levels of psychiatric symptoms, i.e., depression, anxiety, and substance use are associated with CRP concentrations in the blood after adjusting for important medical, social, and demographic covariates in a large sample undergoing screening for several transdiagnostic research studies. Support for an association between CRP and psychiatric symptoms would provide evidence that peripheral inflammation tracks symptom severity. While a major portion of this study is focused on depression, we also wanted to investigate the relationship between CRP and other psychiatric phenotypes such as anxiety and substance use given previous reports linking anxiety and substance use with inflammation [20–24]. A multivariate linear regression (LM) approach was used to determine whether individual level predictions about CRP concentrations could be made.

## Methods

### Participants

This study was approved by the Western Institutional Review Board, written informed consent statement was obtained for each participant, and all study procedures were carried out in accordance with the principles expressed in the Declaration of Helsinki. Two subjects were excluded based on transgender self-report.

A total of 2,532 participants completed an in-person screening visit at the Laureate Institute for Brain Research (LIBR) between 7/1/2016 and 1/30/2019 to determine eligibility for various studies at LIBR. Out of a total of 2,532 participants, 1,724 participants (female: *n* = 1086; male: *n* = 638; ages 18–61 years) with complete data were included in the main analysis. The Diagnostic and Statistical Manual of Mental Disorders-5 (DSM-5) classification of participants based on the Mini International Neuropsychiatric Interview (MINI) are as follows: no psychiatric disorder (healthy controls, *n* = 322), anxiety disorder (*n* = 692), major depressive disorder (MDD) (*n* = 1105), post-traumatic stress disorder (PTSD) (*n* = 206), substance use disorder (*n* = 355), other (*n* = 413). The “other” category included bipolar disorder, alcohol use disorder, psychotic disorder, and obsessive-compulsive disorder. Note that some participants had more than one diagnosis and hence the numbers do not add up to 1,724. Demographic and clinical characteristics of the final participants involved in this study are presented in Table 1, with statistics reported as mean (SD) and median (min, max) values. The CONSORT flow diagram for participant inclusion in this work is presented in Fig. S1. An aggregation plot for the fraction of missing values for each variable is available in Fig. S2. Income was the variable with the most missing data among the 2,532 subjects.

**Table 1:** Demographic and clinical characteristics.

	Female	Male	Overall
	(*N* = 1086)	(*N* = 638)	(*N* = 1724)
log.CRP			
Mean (SD)	0.259 (0.460)	0.196 (0.428)	0.236 (0.449)
Median [Min, Max]	0.232 [−0.301, 1.36]	0.161 [−0.301, 1.36]	0.199 [−0.301, 1.36]
age			
Mean (SD)	32.8 (10.3)	34.3 (10.5)	33.4 (10.4)
Median [Min, Max]	31.0 [18.0, 61.0]	33.0 [18.0, 59.0]	32.0 [18.0, 61.0]
Hispanic_Latino			
No	998 (91.9%)	606 (95.0%)	1604 (93.0%)
Yes	88.0 (8.1%)	32.0 (5.0%)	120 (7.0%)
White			
No	246 (22.7%)	133 (20.8%)	379 (22.0%)
Yes	840 (77.3%)	505 (79.2%)	1345 (78.0%)
Black_African.American			
No	921 (84.8%)	562 (88.1%)	1483 (86.0%)
Yes	165 (15.2%)	76.0 (11.9%)	241 (14.0%)
Native_American			
No	889 (81.9%)	536 (84.0%)	1425 (82.7%)
Yes	197 (18.1%)	102 (16.0%)	299 (17.3%)
Asian			
No	1056 (97.2%)	623 (97.6%)	1679 (97.4%)
Yes	30.0 (2.8%)	15.0 (2.4%)	45.0 (2.6%)
other			
No	1062 (97.8%)	620 (97.2%)	1682 (97.6%)
Yes	24.0 (2.2%)	18.0 (2.8%)	42.0 (2.4%)
log.income			
Mean (SD)	4.34 (1.04)	4.31 (1.04)	4.33 (1.04)
Median [Min, Max]	4.56 [0, 5.74]	4.52 [0, 5.74]	4.54 [0, 5.74]
education			
<7 years of school	2.00 (0.2%)	2.00 (0.3%)	4.00 (0.2%)
Junior high school (7th, 8th, 9th)	22.0 (2.0%)	4.00 (0.6%)	26.0 (1.5%)
Some high school (10th, 11th)	34.0 (3.1%)	29.0 (4.5%)	63.0 (3.7%)
High school graduate (including equivalency exam)	157 (14.5%)	126 (19.7%)	283 (16.4%)
Some college or technical school (at least 1 year)	484 (44.6%)	286 (44.8%)	770 (44.7%)
College graduate	279 (25.7%)	147 (23.0%)	426 (24.7%)
Graduate professional training (Masters or above)	108 (9.9%)	44.0 (6.9%)	152 (8.8%)
log.alcohol			
Mean (SD)	0.379 (0.601)	0.519 (0.769)	0.431 (0.671)
Median [Min, Max]	0 [0, 2.79]	0 [0, 3.40]	0 [0, 3.40]
log.caffeine			
Mean (SD)	2.33 (1.09)	2.36 (1.22)	2.34 (1.14)
Median [Min, Max]	2.71 [0, 4.09]	2.81 [0, 4.48]	2.73 [0, 4.48]
nicotine			
Mean (SD)	0.688 (1.66)	1.34 (2.22)	0.929 (1.91)
Median [Min, Max]	0 [0, 10.0]	0 [0, 8.00]	0 [0, 10.0]
bmi			
Mean (SD)	28.0 (6.14)	27.7 (5.23)	27.9 (5.82)
Median [Min, Max]	27.0 [16.1, 48.3]	26.8 [17.1, 52.1]	26.9 [16.1, 52.1]
percent.body.fat			
Mean (SD)	37.1 (9.31)	25.0 (9.26)	32.6 (11.0)
Median [Min, Max]	38.0 [14.6, 55.8]	24.3 [5.90, 51.2]	32.8 [5.90, 55.8]
waist.hips.ratio			
Mean (SD)	0.862 (0.0763)	0.928 (0.0779)	0.886 (0.0833)
Median [Min, Max]	0.860 [0.659, 1.18]	0.927 [0.528, 1.31]	0.884 [0.528, 1.31]
phq.score			
Mean (SD)	9.56 (7.01)	9.08 (7.13)	9.38 (7.06)
Median [Min, Max]	9.00 [0, 27.0]	8.00 [0, 27.0]	8.00 [0, 27.0]
dast.score			
Mean (SD)	1.26 (2.56)	2.38 (3.45)	1.67 (2.97)
Median [Min, Max]	0 [0, 10.0]	0 [0, 10.0]	0 [0, 10.0]
QIDS_SR.sleep.score			
Mean (SD)	2.08 (0.953)	2.01 (1.01)	2.05 (0.975)
Median [Min, Max]	2.00 [0, 3.00]	2.00 [0, 3.00]	2.00 [0, 3.00]
CRP.detection.range			
LOD (0.5–23)	870 (80.1%)	549 (86.1%)	1419 (82.3%)
LOD (0.8–20)	216 (19.9%)	89.0 (13.9%)	305 (17.7%)

Table 1 describes demographic and clinical characteristics for the 1724 participants as well as the female-only (*n* = 1086) and male-only (*n* = 638) samples. Note. QIDS-SR Quick Inventory of Depressive Symptomatology-self report.

^a^10-based log-transformation was applied. QIDS-SR, Quick Inventory of Depressive Symptomatology-self report.

### Behavioral and demographic measures

Subsequent to phone screening, i.e., during their in-person visits, participants completed a demographics questionnaire, the Patient Health Questionnaire 9 (PHQ-9) [25], the Drug Abuse Screening Test (DAST-10) [26, 27], and the Overall Anxiety Severity and Impairment Scale (OASIS) [28] to measure depression, substance use disorder, and anxiety, respectively. PHQ-9 and OASIS were positively correlated (*r* = 0.79) and showed similar results (Supplementary Fig. S20), therefore we chose to focus on PHQ-9 and DAST. Age, sex, race, ethnicity, annual family income, and education level data were obtained from a self-reported demographics questionnaire. Alcohol and caffeine use were defined according to weekly usage, and nicotine use was measured using the Fagerstrom Test For Nicotine Dependence (FTND) [29]. Sleep disturbance over the prior 7 days was measured using the 4-item Quick Inventory of Depressive Symptomatology-Self Report (QIDS-SR), which was generated with the following items: falling asleep, sleep during night, waking too early, and sleeping too much [30].

The categorical variables, sex, race and ethnicity, immunoassay range, and medication use were binary coded with a 0/1 coding scheme, and education levels were encoded with a value from 1 to 7 sequentially based on the Hollingshead Four-Factor Index of Socioeconomic Status (SES): Subject’s Educational Status. The immunoassays here refer to the detection range as the “CRP Detection Range” (last item) in Table 1. Some subjects’ CRP levels are measured with a different detection range, and this is binary coded.

### Obesity indices

Body mass index (BMI) was calculated based on weight/height during the medical history. Percent Body Fat (PBF) was assessed using an InBody370 Impedance Body Composition Analyzer (InBody Co., Ltd., South Korea). This device uses 15 impedance measurements (3 frequencies: 5 kHz, 50 kHz, 250 kHz; five body segments: right arm, left arm, trunk, right leg, left leg) to produce highly accurate composition estimates and has been found to have a high correlation of 0.99 to dual-energy X-ray absorptiometry (DEXA) for lean body mass in a population of normal-weight adults [31]. Waist-to-hip ratio (WHR) was calculated with waist and height measurements.

### C-reactive protein

CRP was measured using venous whole blood with the Diazyme high sensitivity (hs) CRP point of care (POC) test kit (#DZ135B-SMA-discontinued), a latex enhanced immunoturbidimetric assay on the SMART 700/340 Analyzer (Diazyme Laboratories). CRP levels outside of the immunoassay detection range were truncated to the assay’s upper or lower limit value (Fig. S3). The assays utilized in this study have similar detection ranges, (0.5–23; *n* = 1419) and (0.8–20; *n* = 305) as visualized using raw (Fig. S3A) and log-transformed (Fig. S3B) CRP levels. 370 out of 1419 subjects are below 0.5 mg/L, and 128 out of 305 subjects are below 0.8 mg/L. Initially, 8 subjects were used to test the validity of the POC measures using two separate fingersticks and one IV blood draw for comparison to the Mesoscale Discovery CRP Kit using serum (#K151STD-1).

Additionally, to reduce the complexity of the irreversible compression and information loss once continuous variables were transferred to categorical variables (regression issue), CRP levels were dichotomized based on the median in each dataset (Table S1). We used the median as the threshold to avoid unbalanced datasets. We obtained similar results from both linear regression and logistic regression (using the dichotomized CRP classification, i.e., low vs. high).

### C-reactive protein daily and yearly pattern

Daily and yearly cyclical variables were analyzed for association with CRP. Subjects were partitioned into different groups, based on time of day (hours) or year (months) when CRP was measured. The distribution of log-CRP versus sample collection time (Fig. S4) or month (Fig. S5) were visualized with violin plots.

### Medication use

Medication use within the 2 weeks prior to CRP measurement was extracted from the database, and specific medication classes of interest were included in analysis (Table S2). Serotonin-selective reuptake inhibitors (SSRIs) (11.0%), non-steroidal anti-inflammatory drugs (NSAIDs) (10.0%), contraceptives (9.5%), antihistamines (8.8%), and non-SSRI antidepressants (bupropion, mirtazapine, trazadone, vilazodone, and vortioxetine) (7.9%) were the most widely used medications. Medication use was also classified using dichotomized CRP levels (Table S3).

### Statistical analysis

Nested cross-validation was used to search for optimal model hyper-parameters and obtain a robust and unbiased evaluation of model performance (Fig. S13). Linear and non-linear models, including multivariate linear regression (LM), Principal Component Regression (PCR), Random Forest (RF), and Support Vector Machine (SVM), were used to capture the relationship between log-CRP and variables of interest, and the prediction performances were measured using R-squared (Fig. S14). Among these four models, RF showed the highest R-squared value. The performance of LM was close to RF, and the results obtained from LM and RF (Figs. S15–S19) agreed well with each other in general. Due to the ease of interpretation [32], results from LM were emphasized as the primary findings in this paper. For LM, all the numerical variables were centered and scaled in a pre-processing step to obtain the standardized beta coefficients, which were utilized as the measure of the effect size for each variable.

Regression diagnostics were implemented to verify the linear regression assumptions were not violated and our conclusions were not biased. The residual refers to the difference between model prediction and true observation. The Residual vs. Fitted plot was used to test the assumption of linearity between predictors and outcome by identifying whether residuals presented any non-linear patterns with respect to the fitted values (Fig. S6A–C). The Quantile-Quantile (Q-Q) plot examined the normality of residuals (Fig. S7A–C), and the Residuals vs. Leverage plot was used to identify any outliers which would potentially have significant effects on the model fitting (Fig. S8A–C). The independence assumption (autocorrelation) was also tested using the Durbin Watson Test to examine whether errors were autocorrelated with themselves (Table S4). Lastly, the Variance Inflation Factor (VIF) was calculated to test multicollinearity (Table S5).

First, we tested the unadjusted associations between CRP and PHQ-9 as well as between CRP and DAST. Second, we performed multivariate linear regression using all 36 covariates to determine whether the unadjusted associations between CRP and PHQ-9/DAST would hold. Next, to identify the individual variables weakening the adjusted models, we used the unadjusted beta coefficients from the LM (PHQ-9 and DAST) as the baseline and investigated the change in new beta coefficients when adding other individual variables into the regression model.

## Results

### Pearson correlation for CRP and covariates

**Fig. 1:**
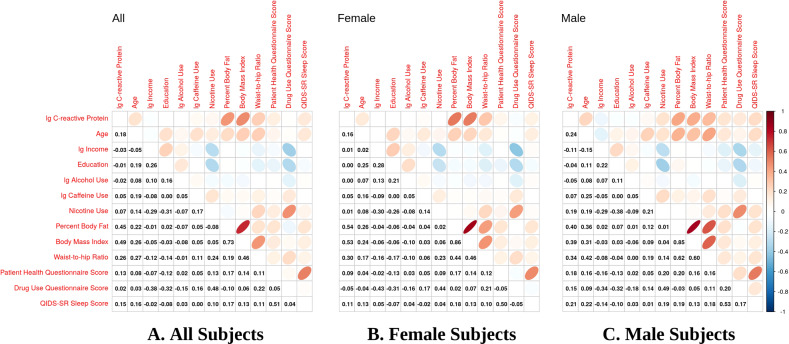
Pearson correlation coefficient plots for CRP and covariates. The plots show the Pearson correlation coefficients for **A** the combined male and female sample (All), **B** female-only sample, and **C** male-only sample. The ellipses are the visualizations for the corresponding Pearson correlation coefficients. Positive correlation is displayed in blue with slope of 1 and negative correlation is in red color with slope of −1. The color intensity and the eccentricity of the ellipse are proportional to the absolute value of correlation coefficient. For Pearson correlation coefficients of 0.1 or greater, CRP was positively correlated for **A** All subjects with age (*r* = 0.18), caffeine use (*r* = 0.05), nicotine use (*r* = 0.07), body mass index (BMI) (*r* = 0.49), percent body fat (PBF) (*r* = 0.45), waist-to-hip ratio (WHR) (*r* = 0.26), PHQ score (*r* = 0.13), and QIDS-SR Sleep score (*r* = 0.15); **B** Female subjects with age (*r* = 0.16), caffeine use (*r* = 0.05), BMI (*r* = 0.53), PBF (*r* = 0.54), and WHR (*r* = 0.30), PHQ (*r* = 0.09), and QIDS-SR (*r* = 0.11); and **C** Male subjects with age (*r* = 0.24), caffeine use (*r* = 0.07), nicotine use (*r* = 0.19), BMI (*r* = 0.39), PBF (*r* = 0.40), WHR (*r* = 0.34), PHQ score (*r* = 0.18), DAST score (*r* = 0.15), and QIDS-SR Sleep score (*r* = 0.21).

To demonstrate the relationship between all continuous variables of interest, Pearson correlation coefficients were calculated and plotted for all subjects, females, and males, separately, specifying a cut point threshold of 0.1 (Fig. 1). In the combined male and female sample (All), CRP was positively correlated (Fig. 1A) with age (*r* = 0.18), caffeine use (*r* = 0.05), nicotine use (*r* = 0.07), body mass index (BMI) (*r* = 0.49), percent body fat (PBF) (*r* = 0.45), waist-to-hip ratio (WHR) (*r* = 0.26), PHQ score (*r* = 0.13), and QIDS-SR Sleep score (*r* = 0.15). In females (Fig. 1B), CRP was positively correlated with age (*r* = 0.16), caffeine use (*r* = 0.05), BMI (*r* = 0.53), PBF (*r* = 0.54), WHR (*r* = 0.30), PHQ score (*r* = 0.09), and QIDS-SR (*r* = 0.11). In males (Fig. 1C), CRP was correlated with age (*r* = 0.24), caffeine use (*r* = 0.07), nicotine use (*r* = 0.19), BMI (*r* = 0.39), PBF (*r* = 0.40), WHR (*r* = 0.34), PHQ score (*r* = 0.18), DAST score (*r* = 0.15), and QIDS-SR Sleep score (*r* = 0.21).

Additional analysis with the depressed group only (Fig. S9), defined as a PHQ-9 score > 9 (*n* = 787), indicated that for the All sample, CRP was correlated with age (*r* = 0.14), nicotine use (*r* = 0.05), BMI (*r* = 0.51), PBF (*r* = 0.45), WHR (*r* = 0.30), and QIDS-SR (*r* = 0.05). Individual symptom items for the All sample (Fig. S10) indicated that CRP was correlated with question (Q)4 (*r* = 0.06) and Q5 (*r* = 0.08), and negatively correlated with Q8 (*r* = −0.05) and Q9 (*r* = −0.08).

### Linear and logistic regression models for simple and entire datasets and association with CRP

To investigate if psychiatric symptoms were associated with CRP levels, we applied both linear and logistic regression using three approaches. First, we tested the direct associations between CRP and PHQ-9 as well as between CRP and DAST (unadjusted/simple models). Second, we ran adjusted/entire models with all potential confounders. Third, we evaluated the effect of each of the 36 covariates individually on the unadjusted associations between CRP and PHQ-9/DAST. For the unadjusted models, the results from linear and logistic regressions were similar (Table 2A). PHQ-9 was significantly associated with CRP levels in the All, Female, and Male samples (All; *β* = 0.125; *β* = 0.252), female-only sample (*β* = 0.091; *β* = 0.171), and male-only sample (*β* = 0.154; *β* = 0.284). In contrast, CRP was only significantly associated with DAST in the male-only sample (*β* = 0.120; *β* = 0.183). In the fully adjusted models, PHQ-9 was not significantly associated with CRP levels in the All, female-only, nor male-only samples, while DAST was significantly associated with CRP in the female-only participants (*β* = −0.088; *β* = −0.228) (Table 2B).

**Table 2:** Linear and Logistic Regression for Simple and Entire Models and Association with CRP.

2 A. Simple Model		PHQ-9		DAST	
		beta	*p*-value	beta	*p*-value
Linear Regression	All	0.125	*0.0000002*	0.012	0.564
	Female	0.091	*0.003*	−0.048	0.115
	Male	0.154	*0.0001*	0.120	*0.003*
Logistic Regression	All	0.252	*0.0000003*	0.033	0.495
	Female	0.171	*0.005*	−0.061	0.324
	Male	0.284	*0.0006*	0.183	*0.027*
2B. Entire Model		PHQ−9		DAST	
		beta	*p*-value	beta	*p*-value
Linear Regression	All	−0.0040	0.872	−0.027	0.305
	Female	−0.034	0.248	−0.088	*0.004*
	Male	0.009	0.833	0.044	0.324
Logistic Regression	All	0.055	0.397	−0.067	0.329
	Female	−0.060	0.497	−0.228	*0.014*
	Male	0.073	0.505	0.100	0.382

We performed linear and logistic regression using two different models, where one model included only PHQ-9 and DAST (simple, unadjusted model) and the other included all 36 variables. Beta Coefficients are reported. (A) For the unadjusted dataset, we analyzed the association between CRP and PHQ-9 and between CRP and DAST. PHQ-9, but not DAST, was significantly associated with CRP in unadjusted/simple models in the (All; *β* = 0.125; *β* = 0.252) and female-only sample (*β* = 0.091; *β* = 0.171). However, for the male-only sample, both PHQ-9 (*β* = 0.154; *β* = 0.284) and DAST (*β* = 0.120; *β* = 0.183) showed significance in unadjusted models. (B) For the All and male-only samples, PHQ-9 and DAST were not associated with CRP after adjusting for the covariates of interest. In the female-only sample, DAST (*β* = −0.088; *β* = −0.228) but not PHQ-9 was significantly associated with CRP in the adjusted/entire model.

Additionally, we looked at the 9 individual items from the PHQ-9 using the same approach (Table S6). Our analysis showed that for the overall sample (*β*: min: −0.03; max: 0.02) and male-only sample (*β*: min: −0.05; max: 0.04) there were no significant relationships between CRP and any of the individual PHQ-9 items in the fully adjusted model (*p*-values > 0.20; results not shown). For females, only item-3 (trouble falling or staying asleep, or sleeping too much) and item-9 (thoughts that you would be better off dead, or of hurting yourself) showed marginal significance (*β* = −0.059; *p* = 0.054) and (*β* = −0.053; *p* = 0.04), respectively.

### Multivariate linear regression and association of CRP with covariates

**Fig. 2:**
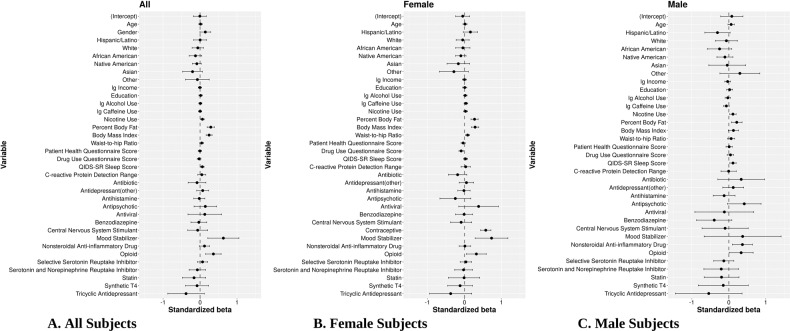
Standardized beta coefficients and association between CRP and all covariates in multivariate linear regression. Standardized beta coefficients and corresponding 95% confidence intervals are depicted as dots and error bars. **A** Significant variables for the combined male and female sample (All) included mood stabilizer and opioid use, PBF, BMI, nicotine use, male sex, and QIDS-SR sleep score. **B** Additional significant variables for the female-only sample included oral contraceptive use (OC use), WHR, and DAST score. **C** Additional significant variables for male-only sample included NSAID use.

To determine which factors were associated with CRP in the adjusted model with potential confounders, standardized beta coefficients and corresponding 95% intervals were obtained from LM on three different samples, i.e., all subjects (Fig. 2A), female subjects (Fig. 2B), and male subjects (Fig. 2C). For All subjects, LM indicated that mood-stabilizer treatment (*β* = 0.630, *p* = 0.003), opioid use (*β* = 0.360, *p* = 7.23E-4), PBF (*β* = 0.289, *p* = 5.35E-9), and BMI (*β* = 0.244, *p* = 4.12E-9) were associated with increased CRP concentrations and had the largest effect sizes. Additionally, male sex (*β* = 0.142, *p* = 0.056), nicotine use (*β* = 0.063, *p* = 0.012) and QIDS-SR (*β* = 0.061, *p* = 0.013) showed smaller but significant effects. Oral contraceptive (OC) use (*β* = 0.576, *p* = 2.06E-15), WHR (*β* = 0.086, *p* = 0.003), and DAST score (*β* = −0.088, *p* = 0.004) were variables associated with CRP in females. NSAID use (*β* = 0.367, *p* = 0.008) was significant for males only.

Variables that did not achieve statistically significant associations with CRP in the adjusted model included depressive symptoms (PHQ-9) (*β* = −0.004, *p* = 0.872), age (*β* = 0.013, *p* = 0.587), log-income (*β* = −0.005, *p* = 0.837), education (*β* = 0.015, *p* = 0.523), SSRI use (*β* = 0.066, *p* = 0.346), SNRI use (*β* = −0.073, *p* = 0.517), antihistamine (*β* = −0.021, *p* = 0.784), antipsychotic (*β* = 0.142, *p* = 0.362), antibiotic (*β* = −0.083, *p* = 0.501), antiviral (*β* = 0.127, *p* = 0.584), benzodiazepine (*β* = −0.033, *p* = 0.759), statin type (β = −0.164, *p* = 0.308), log-caffeine (*β* = 0.001, *p* = 0.952), log-alcohol (*β* = 0.008, *p* = 0.718), Hispanic/Latino (*β* = 0.002, *p* = 0.986), Black/African American (*β* = −0.124, *p* = 0.153), White (*β* = −0.061, *p* = 0.437), Native American (*β* = −0.088, *p* = 0.155), Asian (*β* = −0.204, *p* = 0.136), and other (*β* = −0.072, *p* = 0.657).

Additional analysis with the depressed group only (Fig. S11), indicated that for the All sample, CRP was associated with nicotine use (β = 0.07, *p* = 0.05), PBF (*β* = 0.284, *p* = 9.38E-5), BMI (*β* = 0.277, *p* = 1.26E-5), mood stabilizer use (*β* = 0.586, *p* = 0.036), and opioid use (*β* = 0.308, *p* = 0.039).

### Sex and obesity indices and association of CRP with covariates

**Fig. 3:**
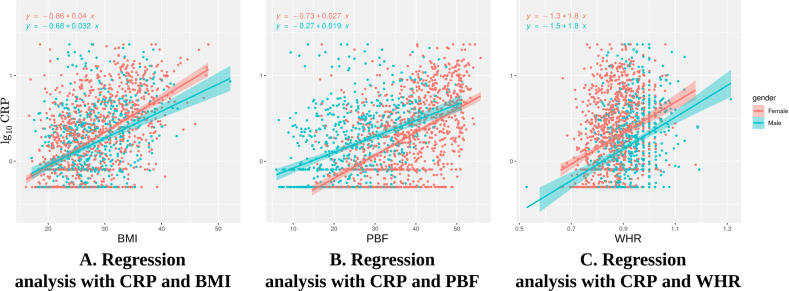
Linear regression plots for C-reactive Protein (CRP) versus Body Mass Index (BMI), Percent Body Fat (PBF), and Waist-to-Hip Ratio (WHR). The plots show the effect of the potential confounding variable [sex—female (pink); male (green)] on the regression analysis of the relationship between CRP and obesity indices (BMI, PBF, and WHR). **A** The regression plot shows that when controlling for BMI, CRP is higher in females compared to males. **B** The regression plot shows that when we control for PBF, CRP is higher in males compared to females. **C** The regression plot shows that when we control for WHR, CRP is higher in females compared to males.

According to the results from multivariate linear regression, males had higher CRP than females after controlling for the other covariates. However, the Welch Two Sample *t*-test showed significantly higher CRP in females than males (female: mean = 0.259, male: mean = 0.196; *t* = 2.866; df = 1414.2; *p* = 0.004). We found the differences were accounted for by using different obesity indices. Figure 3 shows the influence of BMI, PBF, and WHR on CRP for males and females. When controlling for BMI, female sex was associated with higher CRP as compared to male sex (Fig. 3A). However, controlling for PBF showed that male sex was associated with higher CRP than female sex (Fig. 3B). Lastly, controlling for WHR showed that female sex was associated with higher CRP than male sex (Fig. 3C).

Additional analysis with the depressed group only (Fig. S12), indicated that for the All sample, the main variables impacting the relationship between CRP and depressive symptoms included: PBF (*β* = 0.202, *p* = 2.04E-5), BMI (*β* = 0.306, *p* = 5.55E-9), and WHR (*β* = 0.123, *p* = 0.001).

### Confounder identification on association with CRP and PHQ-9/DAST

To identify the variables that modulate the association between CRP and psychiatric symptoms, we used beta coefficients from the unadjusted linear regression model (only PHQ-9 and DAST) as the baseline. After progressively adding one new variable into the model, we compared these new beta coefficients for PHQ-9 and DAST with the baseline. Table 3 shows both linear and logistic regression models, which indicated that an unadjusted model including only PHQ-9 or DAST was significantly associated with CRP. For PHQ-9, several variables [All (PBF, BMI, WHR, and QIDS-SR), Female (PBF, BMI, WHR, and QIDS-SR), and Male (age, PBF, BMI, WHR, and QIDS-SR)] affected CRP concentrations. For DAST, [All (nicotine use, PBF, BMI, and WHR), Female (nicotine use, PBF, BMI, WHR, and contraceptive use), and Male (nicotine use, PBF, WHR)] affected CRP concentrations.

**Table 3:** Effect of Selected Confounders on Association with CRP and PHQ-9/DAST.

	PHQ-9	Association	beta	DAST	Association	beta
All	None		0.124	None		0.014
	Percent Body Fat	Decrease	0.045	Nicotine Use	Increase	−0.017
	Body Mass Index	Decrease	0.057	Percent Body Fat	Increase	0.064
	Waist-to-Hip Ratio	Decrease	0.099	Body Mass Index	Decrease	−0.010
	QIDS-SR	Decrease	0.065	Waist-to-Hip Ratio	Increase	−0.042
Female	None		0.091	None		−0.048
	Percent Body Fat	Decrease	−0.002	Nicotine Use	Increase	−0.062
	Body Mass Index	Decrease	0.016	Percent Body Fat	Increase	−0.061
	Waist-to-Hip Ratio	Decrease	0.050	Body Mass Index	Increase	−0.089
	QIDS-SR	Decrease	0.047	Waist-to-Hip Ratio	Increase	−0.117
				Contraceptive Use	Decrease	−0.030
Male	None		0.154	None		0.120
	Age	Decrease	0.122	Nicotine Use	Decrease	0.060
	Percent Body Fat	Decrease	0.069	Percent Body Fat	Increase	0.150
	Body Mass Index	Decrease	0.096	Waist-to-Hip Ratio	Decrease	0.095
	Waist-to-Hip Ratio	Decrease	0.109			
	QIDS-SR	Decrease	0.074			

To identify the variables that modulate the association between CRP and psychiatric symptoms, we used beta coefficients from the unadjusted linear regression model (only PHQ-9 and DAST) as the baseline. After progressively adding one new variable into the model, we compared these new beta coefficients for PHQ-9 and DAST with the baseline. Table 3 shows both linear and logistic regression models, which indicated that an unadjusted model including only PHQ-9 or DAST was significantly associated with CRP. For PHQ-9, All (PBF, BMI, WHR, and QIDS-SR), Female (PBF, BMI, WHR, and QIDS-SR), and Male (age, PBF, BMI, WHR, and QIDS-SR)] variables affected CRP concentrations. For DAST, All (nicotine use, PBF, BMI, and WHR), Female (nicotine use, PBF, BMI, WHR, and OC use), and Male (nicotine use, PBF, WHR)] variables affected associations between PHQ-9 or DAST and CRP.

**Fig. 4:**
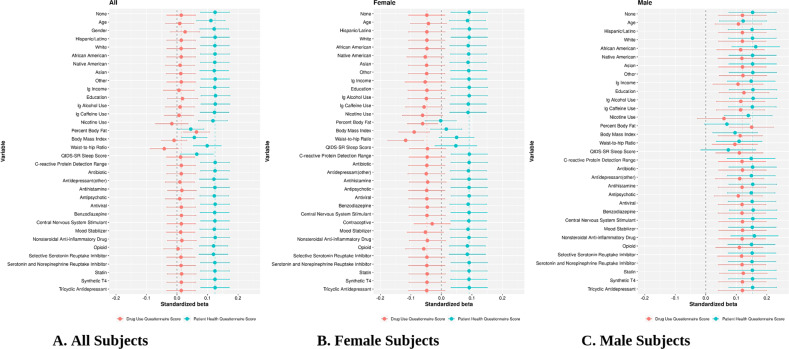
Standardized beta coefficients for confounder effect of variables on association with CRP. Plots show the variables with the largest shifts from baseline with DAST only (pink) or PHQ-9 only (green), indicating a decreased or increased association between CRP and psychiatric symptoms. The labels of the *y*-axis represent which individual variable is included in the model. Plots for **A** All; **B** Female; and **C** Male subjects. Note that the top line of the figure (indicated by None) shows the unadjusted/simple model, and each subsequent line shows the effect of the relevant covariate progressively added on the unadjusted/simple relationship. Initially for the All sample (Fig. 4A), PHQ-9 is significantly associated with CRP in the unadjusted/simple models (as seen by the right shift from baseline of 0), while DAST is not (the error bar crosses the baseline of 0). For PHQ-9, PBF, BMI, and WHR all shift the association to the left, thereby decreasing the beta values for this association. On the other hand, for DAST, PBF shifts the association to the right (increases) while BMI and WHR decrease the beta values for this association, showing opposite effects. For the female-only sample in the unadjusted/simple model, PHQ-9, but not DAST, is significantly associated with CRP (Fig. 4B). Here, PBF, BMI, and WHR decrease these beta values. For the male-only sample (Fig. 4C), both DAST and PHQ-9 are significantly associated with CRP in the unadjusted/simple model, and like the females, PBF, BMI, and WHR decrease this association for PHQ-9. Interestingly, BMI and WHR decrease this association for DAST, while PBF increases this association in males.

In Fig. 4A–C, the beta coefficients and 95% confidence intervals of PHQ-9 and DAST are plotted in green and pink, respectively. Note that the top line of the figure (indicated by None) shows the unadjusted/simple model, and each subsequent line shows the effect of the relevant covariate progressively added on the unadjusted/simple relationship. Initially for the All sample (Fig. 4A), PHQ-9 is significantly associated with CRP (green) in the unadjusted/simple models (as seen by the right shift from baseline of 0), while DAST is not (the error bar crosses the baseline of 0). For PHQ-9, PBF, BMI, and WHR all shift the association to the left, thereby decreasing the beta values for this association. On the other hand, for DAST, PBF shifts the association to the right (increases) while BMI and WHR decrease the beta values for this association, showing opposite effects. For the female-only sample in the unadjusted/simple model, PHQ-9, but not DAST, is significantly associated with CRP (Fig. 4B). Here, PBF, BMI, and WHR decrease these beta values. For the male-only sample (Fig. 4C), both PHQ-9 and DAST are significantly associated with CRP in the unadjusted/simple model, and like the females, PBF, BMI, and WHR decrease this association for PHQ-9. Interestingly, BMI and WHR decrease this association for DAST, while PBF increases this association in males.

## Discussion

This study examined whether depression, anxiety, and substance use-related symptoms were associated with CRP concentrations in the blood after adjusting for relevant medical, social, and demographic covariates. We found that greater BMI, PBF, opioid, mood-stabilizer, and nicotine use, WHR, and sleep disturbance were associated with increased CRP concentrations in both sexes in the fully adjusted model. Female and male-specific analyses also showed that oral contraceptive (OC) use and DAST score for females and NSAID use for males were significantly associated with higher CRP concentrations. However, severity of depression or anxiety, age, education, income, race/ethnicity, SSRI/SNRI, and several other variables were not individually associated with CRP concentrations. While psychiatric symptoms did not achieve significance after adjustment of covariates, a simple unadjusted model showed that PHQ-9 was significantly associated with CRP in all participants, while DAST was only associated in male-only participants. These results provide evidence that the strength of the association between CRP and the severity of psychiatric symptoms depends on which covariates are controlled during statistical analyses.

This study found that several biomedical and health-related variables were associated with CRP including biological sex, anthropometric variables, OC use, and sleep disturbance. Male sex was moderately associated with elevated CRP concentrations, which is contrary to previously reported population-based studies [33, 34]. On the other hand, regression plots showed that female sex was associated with higher CRP concentrations when controlling for BMI or WHR, and a univariate analysis also confirmed a higher CRP mean concentration in females versus males. Interestingly, our results showed that male sex was associated with higher CRP concentrations when controlling for PBF, which many studies fail to do, instead only adjusting for BMI [35, 36]. Theoretical explanations have arisen for the association of biological sex and elevated CRP concentrations including (1) greater adiposity in women, where adipose tissue is more metabolically active, leads to increased production of IL-6 and subsequently CRP [37–39]; (2) the amount of total body fat rather than where the fat is distributed in the body, for example, women have higher subcutaneous fat deposits and males have greater intra-abdominal visceral fat deposits [40, 41]; and (3) sex steroids, estrogen and testosterone, which may be impacted by steroid receptor expression in both subcutaneous and visceral adipose tissue [42, 43].

Interpretations on biological sex and association with CRP rely heavily on obesity indices including BMI, PBF, and WHR, which were also associated with CRP concentrations in this study and tend to vary in women and men [36, 40, 44, 45]. BMI is especially important because studies have shown a small association between CRP and depression when BMI is adjusted, but studies not adjusting for BMI have shown an effect size three times as large [13, 38]. It is hypothesized that inflammation in obesity results from metabolic disturbances in adipose tissue leading to increases in cytokine production and inflammatory pathway activation [38, 46]. CRP concentrations were shown to be significantly influenced by adiposity and consistently higher in women but more variable in men as measured by BMI, total fat mass, truncal fat, lower body fat, and/or subcutaneous fat mass [39, 40]. A Taiwanese-based population study identified PBF mass as a significant factor associated with high levels of CRP in both women and men after adjusting for confounding factors, while WHR was only associated with CRP in women [45]. Obese women (6.21 times) and men (2.13 times) were more likely to have WHR positively associated with elevated and clinically raised CRP levels compared to normal-weight counterparts and explained the highest percentage of the variability of CRP in men [47].

Here, we identified the main variables impacting the relationship of CRP with depressive (PHQ-9 score) and drug use (DAST) symptoms were PBF, BMI, and WHR. During our confounding analysis, all three variables decreased the relationship with CRP and depressive symptoms. Qin et al., found the CRP-depression relationship to no longer be significant in participants with BMI groups that ranged from normal to obesity [48]. Surprisingly, for the DAST, BMI and WHR decreased the association with CRP, but PBF increased this association in males, which is consistent with our results indicating that males have higher CRP than females when controlling for PBF as shown in other studies [49, 50]. To this, one study suggested that PBF was more strongly correlated with fat content than BMI, which may result in increased inflammation from fat stores [51]. Unraveling the intricacies of sex differences are complicated, and more studies are needed to identify key biological mechanisms for these findings.

Another critical variable that can confound associations with CRP is OC use. In this study, OC use was associated with elevated CRP in females as demonstrated by others [52–55], and in an independent sample of subjects with mood disorders [56]. Other research data showed that CRP plasma levels were 2 times [57] and 3 times higher [58] in OC users than in non-OC users. Additionally, OC use was the strongest predictor of low grade inflammation, CRP (>3 mg/L below or equal to 10 mg/L), in pre-menopausal women [59], and, OC use predicted 32% of variance in CRP concentrations in young, healthy, non-smoking, non-obese women [57]. The mechanism through which OC use increases CRP is not well understood, but mechanisms proposed for this association include: 1) a metabolic rather than an inflammatory response as several studies have reported increases in CRP concentrations in the absence other proinflammatory molecules such as IL-6 and TNF, which is related to a direct effect on hepatocyte CRP synthesis; 2) OC-mediated hypomethylation of DNA leading to increase expression of the CRP gene in monocytes; and 3) lower insulin sensitivity produced by progestins with direct action on pancreatic beta cells [57, 60–63]. Our findings suggest that several biomedical and health-related variables are associated with low grade inflammation as defined by CRP levels. Female sex is likely associated with elevated levels of inflammation, including CRP, only when controlling for BMI or WHR, which could be explained by several mechanistic theories. This may also indicate that conclusions on sex differences in CRP and other inflammatory measures must be reported carefully and with caveats based on which confounding variables are controlled for.

Here, we also demonstrated that opioid, mood stabilizer, nicotine, and NSAID (males only) use, and sleep disturbance were also associated with elevated CRP concentrations, which is consistent with other studies [64–68]. A previous cross-sectional analysis using LM showed CRP levels were higher in opioid users, while controlling for demographic and clinical factors [69]. Both central and peripheral opioid receptors contribute to early stages of the inflammatory response attributed to opioid receptors on a variety of immune cells including blood mononuclear cells, T and B cells, monocytes, and macrophages [70, 71]. Opioid agonists used for analgesic therapy, such as morphine and fentanyl, have also been shown to be proinflammatory through Toll-like receptor 4 (TLR4) signaling [23, 72], which has been reported to mediate CRP-induced effects through the p38 MAPK pathway in vascular smooth muscle cells [73]. The use of opioids for pain also extends into surgical arenas, where post-operative CRP levels are also increased and are positively associated with opioid consumption and higher pain scores [74]. In addition to opioid use, our study also found a positive association with elevated CRP concentrations and mood stabilizer use—but in a small sample of 17 subjects within a 2-week period before CRP measurement. Therefore, these results should be treated with caution. Several studies have reported no association between mood stabilizer use (lithium, valproic acid, and lamotrigine) and CRP concentrations [75, 76]. There are at least three possible interpretations of our finding. First, individuals with mood disorders who receive augmentation treatment with mood stabilizers may represent a more severe and possibly treatment resistant group. Second, the mechanism of action of mood stabilizers, which includes alteration of neuronal excitability and activation of intracellular signaling cascades associated with cell survival, growth, and metabolism [77] may contribute to inflammation. However, different mood stabilizers are thought to work via different mechanisms including regulation of the immune system, oxidative stress pathways, and glycogen synthase kinase (GSK)-3β, [65] and this study was not powered to disentangle the effects of specific types of mood stabilizers. Third, those who took mood stabilizers were mostly female subjects (1.3%) compared to male subjects (0.5%) indicating that sex may have influenced our finding.

Nicotine use and sleep disturbance showed smaller but significant associations with CRP in this study as previously demonstrated by other research groups [66, 78–80]. Nicotine exerts its effects through activation of the nicotinic acetylcholine receptor [81] and nicotine produces proinflammatory mediators such as TNF and IL-6 in in vitro and mouse models through the NF-κB transcription factor [82]. That said, the effects of nicotine are complicated as its direct pharmacological effect may be anti-inflammatory [83]. The proinflammatory effects most likely arise from secondary factors such as tissue damage and the fact that cigarettes contain other proinflammatory chemicals [84]. Sleep disturbance also showed a significant but small effect in this study, consistent with other studies showing that poor sleep quality and short sleep duration are associated with higher CRP concentrations [67, 85, 86]. Evidence suggests that sleep and the immune system can have bi-directional effects on each other with sleep promoting cytokine expression and cytokines influencing sleep and sleep depth [87, 88]. Poor sleep quality, poor sleep continuity, and short sleep duration has been linked to adverse health outcomes and exaggerated inflammatory responses including increases in TNF, IL-6, and CRP. Conceivably, sleep disturbance could increase inflammation-mediated effects in psychiatric disorders, as indicated by increasing concentrations of CRP through inflammatory and depressive pathways.

NSAID use was also associated with elevated CRP concentration but in males only. One meta-analysis of randomized control trials in rheumatoid arthritis showed that NSAIDs can modulate CRP levels, which may be dependent on the drug’s mechanism of action [89]. Other studies have also shown significant increases in CRP levels 72 h after treatment with NSAIDs post molar extraction surgery [68, 90]. This would suggest that people taking NSAIDs are more likely to have inflammation. NSAID-specific sex effects were not analyzed in these studies, but cyclooxygenase genes, the molecular targets for NSAIDs, may work differently in males and females to influence sex-specific inflammatory effects [91].

Interestingly, supplementary chi-squared analyses showed that mood stabilizer, NSAID, and opioid use were significantly higher in the high versus low CRP dichotomized groups (Table S3). Taken together, our finding suggests that the use of opioids, mood stabilizers, nicotine, NSAIDs and sleep disturbance in psychiatric patients may be predictive of inflammatory-mediated effects through mechanisms in the central nervous system and periphery.

The variables that showed non-significant associations with CRP included self-reported mood, age, education, income, SSRI use, and daily/yearly pattern in this study. While meta-analyses and numerous individual studies have reported increased concentrations of inflammatory markers, including CRP in depressed subjects, it is difficult to control for the full array of demographic and lifestyle variables that may confound this association. In some cases, prospective data are weak and most meta-analyses of depression and CRP include studies in which the vast majority have not considered health confounds as done here. The Horn et al. (2018) meta-analysis concluded that associations between CRP and depression may be inflated if rigorous and higher methodological standards are not followed and that only 8 studies with continuous predictors qualified for the most rigorous stage of their meta-analysis (*n* = 78 total) [18]. Other studies have also shown no significant association between depression and CRP after adjusting for potential confounders including BMI and smoking [67, 80, 92]. CRP was shown not to be related to depression when looking at MDD, but only increased in a subtype of depression with increased appetite [93].

Along these lines, additional analysis looking at depressed samples only, did not reveal any changes as compared to our analysis with the entire study population in the adjusted model. On the other hand, our unadjusted model did show that PHQ-9 was significantly associated with CRP levels (*β* = 0.125) and our effect size was comparable to that reported in the UK Biobank study of depression and CRP (*β* = 0.144) by *Pitharouli and colleagues* [9].

Additionally, while we found no significant association with the nine individual items for PHQ-9 with linear regression for all and male-only participants, there was marginal significance for item-3 and item-9 in female-only participants. While the PHQ-9 reflects the nine major criteria for major depression, items 3 and 9 focus on sleep and suicidality, respectively [94]. Interestingly, sleep disturbance, based on the QIDS sleep score, was also significantly associated with CRP in females, which we found to be highly correlated with Item-3. Item-9 (suicidality) also showed marginal significance with CRP in females. This finding was not surprising as suicidality has previously been associated with inflammation [95, 96].

Consist with our results, other studies have reported no association between CRP and socio-demographic factors such as age, education, and income [80, 97]. However, in other studies examining the association between CRP and age, CRP concentrations are reported to increase in an age-dependent manner and are even higher in aging populations with underlying medical conditions [98–100]. Ages in our study ranged from 18 to 61, which could be one reason why there was no association between CRP and age. Several studies have reported that education is inversely related to CRP after adjusting for demographic, clinical, and behavioral factors such as age, sex, BMI, and smoking [97, 101]. However, other studies have also reported no association of CRP and income [80]. Additionally, we did not find an association between CRP and SSRI use. Hamer et al. reported an association between SSRI use and CRP, although this was largely confounded by smoking, and Dawood et al. reported that SSRI use increased CRP concentrations in MDD patients in a within subject design [102, 103]. Conversely, O’Brien et al. details two studies (between-subjects and within-subject designs) where CRP concentrations did not differ between medicated depressed participants and healthy controls and second, CRP concentrations significantly decreased after SSRI treatment [104]. Lastly, there was no association between CRP and daily or yearly pattern as reported in other studies [105, 106]. Baseline CRP levels were stable over 24 h and not subject to time-of-day variation [105]. Conversely, one research group found a significant variation of CRP serum levels, with highest levels occurring in the morning and lowest at midday [107].

The current study has several limitations. First, the sample was comprised of healthy volunteers, participants with mood and anxiety disorders as well as participants with substance use disorders. Although consistent with the demographics of the local catchment area, these results may need replication in other study populations. Second, while the PHQ-9 does not probe as many symptoms as other scales, it has been shown to be a valid and reliable measure of general depressive symptoms and correlates highly with other self-report and clinician-administered scales [25, 94, 108]. Third, the sleep assessment was based on one subjective item in the QIDS-SR and is not a dedicated objective measure of sleep, such as actigraphy. Nevertheless, it has been used in other research studies to measure sleep quality [80, 109].

While several limitations have been mentioned, this investigation also has several strengths. First, the sample size was large and representative of a diverse community population. Second, we performed multiple statistical analysis including LM and RF, which showed similar results when PHQ-9 or OASIS were analyzed as the independent variable predictor. Lastly, while we saw that PHQ-9 and CRP were significantly associated using a simple unadjusted model, unlike other studies showing associations with CRP and sex after adjusting for BMI only, our study adjusted for all anthropometric variables, BMI, PBF, and WHR, which attenuated the association of CRP and female sex with PBF regression.

## Conclusion

This study reported several biomedical and health-related variables to be positively associated with CRP including BMI, PBF, and opioid, mood stabilizer, and OC use. WHR, nicotine use, and sleep disturbance also had significant but smaller effects. After adjustment for covariates, PHQ-9 and DAST were no longer associated with CRP, which was determined to be attributable to several confounders such as PBF, BMI, WHR, QIDS-SR, and age (PHQ-9) and PBF, BMI, WHR, nicotine and contraceptive use (DAST). We should be cautious in attributing inflammation to depression per se, since there are multiple factors that co-occur with depression that appear to contribute to inflammation.

## Supplementary information

Supplemental Materials

## Data Availability

The code used to generate manuscript’s data can be accessed at the following link: https://github.com/nidaye1999/CRP.
