# Supplementary material for: Psychiatric symptoms are not associated with circulating CRP concentrations after controlling for medical, social, and demographic factors
Source: Transl Psychiatry. 2022 Jul 12;12:279. doi: 10.1038/s41398-022-02049-y (PMC9276683; doi:10.1038/s41398-022-02049-y)
Supplement: Supplementary file 1 — Supplemental Materials [file 41398_2022_2049_MOESM1_ESM.docx]

**Supplemental Material**

Psychiatric symptoms are not associated with circulating CRP concentrations after controlling for medical, social, and demographic factors.

Leandra K. Figueroa-Hall^1^, Ph.D., Bohan Xu^1,2^, Rayus Kuplicki^1^, Ph.D., Bart N. Ford^3^, Ph.D., Kaiping Burrows^1^, Ph.D., T. Kent Teague^4^, Ph.D., Sandip Sen^2^, Ph.D., Hung-Wen Yeh^5^, Ph.D., Michael R. Irwin^6^, M.D., Jonathan Savitz^1,7*^, Ph.D., Martin P. Paulus^1,7*^, M.D.

1. Laureate Institute for Brain Research, Tulsa OK, 74136

2. Department of Computer Science, Tandy School of Computer Science, The University of Tulsa, OK 74104

3. Department of Pharmacology & Physiology, Oklahoma State University, Center for Health Sciences, Tulsa, OK, 74107

4. Department of Surgery and Department of Psychiatry, University of Oklahoma-School of Community Medicine, Tulsa, OK 74135

5. Division of Health Services & Outcomes Research, Children’s Mercy Kansas City, Kansas City, MO 64108

6. Department of Psychiatry and Behavioral Sciences, UCLA Geffen School of Medicine, Los Angeles, CA 90095

7. Oxley College of Health Sciences, The University of Tulsa, Tulsa, OK 74199

* Equal contributors

Corresponding Author:

Leandra Figueroa-Hall

[lfigueroa-hall@laureateinstitute.org](mailto:lfigueroa-hall@laureateinstitute.org)

Phone: 443-468-7095

| **Table S1. Demographic and Clinical Characteristics classified with dichotomized CRP Levels** | | | |
| --- | --- | --- | --- |
|  | **High CRP**  **lg CRP>0.02** | **Low CRP**  **lg CRP<0.02** | **Overall** |
|  | **(N=860)** | **(N=864)** | **(N=1724)** |
| **lg C-reactive Protein** |  |  |  |
| Mean (SD) | 0.620 (0.288) | -0.147 (0.163) | 0.236 (0.449) |
| Median [Min, Max] | 0.566 [0.201, 1.36] | -0.164 [-0.301, 0.199] | 0.199 [-0.301, 1.36] |
| **Age** |  |  |  |
| Mean (SD) | 35.2 (10.3) | 31.5 (10.2) | 33.4 (10.4) |
| Median [Min, Max] | 34.0 [18.0, 59.0] | 29.0 [18.0, 61.0] | 32.0 [18.0, 61.0] |
| **Gender** |  |  |  |
| Female | 563 (65.5%) | 523 (60.5%) | 1086 (63.0%) |
| Male | 297 (34.5%) | 341 (39.5%) | 638 (37.0%) |
| **Hispanic/Latino** |  |  |  |
| No | 803 (93.4%) | 801 (92.7%) | 1604 (93.0%) |
| Yes | 57 (6.6%) | 63 (7.3%) | 120 (7.0%) |
| **White** |  |  |  |
| No | 199 (23.1%) | 180 (20.8%) | 379 (22.0%) |
| Yes | 661 (76.9%) | 684 (79.2%) | 1345 (78.0%) |
| **African American** |  |  |  |
| No | 736 (85.6%) | 747 (86.5%) | 1483 (86.0%) |
| Yes | 124 (14.4%) | 117 (13.5%) | 241 (14.0%) |
| **Native American** |  |  |  |
| No | 703 (81.7%) | 722 (83.6%) | 1425 (82.7%) |
| Yes | 157 (18.3%) | 142 (16.4%) | 299 (17.3%) |
| **Asian** |  |  |  |
| No | 844 (98.1%) | 835 (96.6%) | 1679 (97.4%) |
| Yes | 16 (1.9%) | 29 (3.4%) | 45 (2.6%) |
| **Other** |  |  |  |
| No | 838 (97.4%) | 844 (97.7%) | 1682 (97.6%) |
| Yes | 22 (2.6%) | 20 (2.3%) | 42 (2.4%) |
| **lg Income** |  |  |  |
| Mean (SD) | 4.29 (1.07) | 4.37 (1.01) | 4.33 (1.04) |
| Median [Min, Max] | 4.54 [0, 5.60] | 4.56 [0, 5.74] | 4.54 [0, 5.74] |
| **Education** |  |  |  |
| Less than seven years of school | 3 (0.3%) | 1 (0.1%) | 4 (0.2%) |
| Junior high school (7th, 8th, 9th) | 14 (1.6%) | 12 (1.4%) | 26 (1.5%) |
| Some high school (10th, 11th) | 35 (4.1%) | 28 (3.2%) | 63 (3.7%) |
| High school graduate (including equivalency exam) | 140 (16.3%) | 143 (16.6%) | 283 (16.4%) |
| Some college or technical school (at least one year) | 375 (43.6%) | 395 (45.7%) | 770 (44.7%) |
| College graduate | 215 (25.0%) | 211 (24.4%) | 426 (24.7%) |
| Graduate professional training (Masters or above) | 78 (9.1%) | 74 (8.6%) | 152 (8.8%) |
| **lg Alcohol Use** |  |  |  |
| Mean (SD) | 0.431 (0.690) | 0.430 (0.652) | 0.431 (0.671) |
| Median [Min, Max] | 0 [0, 3.40] | 0 [0, 2.90] | 0 [0, 3.40] |
| **lg Caffeine Use** |  |  |  |
| Mean (SD) | 2.42 (1.09) | 2.26 (1.18) | 2.34 (1.14) |
| Median [Min, Max] | 2.81 [0, 4.07] | 2.70 [0, 4.48] | 2.73 [0, 4.48] |
| **Nicotine Use** |  |  |  |
| Mean (SD) | 1.08 (2.04) | 0.775 (1.76) | 0.929 (1.91) |
| Median [Min, Max] | 0 [0, 9.00] | 0 [0, 10.0] | 0 [0, 10.0] |
| **Percent Body Fat** |  |  |  |
| Mean (SD) | 36.9 (10.6) | 28.3 (9.54) | 32.6 (11.0) |
| Median [Min, Max] | 38.9 [6.50, 55.8] | 27.9 [5.90, 51.2] | 32.8 [5.90, 55.8] |
| **Body Mass Index** |  |  |  |
| Mean (SD) | 30.3 (6.01) | 25.5 (4.51) | 27.9 (5.82) |
| Median [Min, Max] | 30.0 [16.6, 52.1] | 24.9 [16.1, 40.6] | 26.9 [16.1, 52.1] |
| **Waist-to-hip Ratio** |  |  |  |
| Mean (SD) | 0.905 (0.0817) | 0.867 (0.0805) | 0.886 (0.0833) |
| Median [Min, Max] | 0.905 [0.667, 1.31] | 0.868 [0.528, 1.19] | 0.884 [0.528, 1.31] |
| **Patient Health Questionnaire Score** |  |  |  |
| Mean (SD) | 10.3 (7.03) | 8.50 (6.98) | 9.38 (7.06) |
| Median [Min, Max] | 10.0 [0, 27.0] | 7.00 [0, 27.0] | 8.00 [0, 27.0] |
| **Drug Use Questionnaire Score** |  |  |  |
| Mean (SD) | 1.74 (3.04) | 1.60 (2.90) | 1.67 (2.97) |
| Median [Min, Max] | 0 [0, 10.0] | 0 [0, 10.0] | 0 [0, 10.0] |
| **QIDS-SR Sleep Score** |  |  |  |
| Mean (SD) | 2.17 (0.912) | 1.93 (1.02) | 2.05 (0.975) |
| Median [Min, Max] | 2.00 [0, 3.00] | 2.00 [0, 3.00] | 2.00 [0, 3.00] |
| **C-reactive Protein Detection Range** |  |  |  |
| LOD (0.5-23) | 743 (86.4%) | 676 (78.2%) | 1419 (82.3%) |
| LOD (0.8-20) | 117 (13.6%) | 188 (21.8%) | 305 (17.7%) |

**Table S1. Demographic and Clinical Characteristics classified with dichotomized CRP Levels.** To reduce the complexity of the regression analysis, CRP levels were dichotomized based on the median in each dataset. We obtained similar conclusions from both the logistic and linear regression models.

**Table S2. Participant Medication Use**

|  | Female (N=1086) | Male (N=638) | Overall (N=1724) |
| --- | --- | --- | --- |
| Antibiotic | 44 (4.1%) | 8 (1.3%) | 52 (3.0%) |
| Antidepressant (Other) | 83 (7.6%) | 54 (8.5%) | 137 (7.9%) |
| Antihistamine | 110 (10.1%) | 41 (6.4%) | 151 (8.8%) |
| Antipsychotic | 16 (1.5%) | 18 (2.8%) | 34 (2.0%) |
| Antiviral | 9 (0.8%) | 5 (0.8%) | 14 (0.8%) |
| Benzodiazepine | 56 (5.2%) | 16 (2.5%) | 72 (4.2%) |
| CNS Stimulant | 33 (3.0%) | 8 (1.3%) | 41 (2.4%) |
| Contraceptive | 164 (15.1%) | 0 (0%) | 164 (9.5%) |
| Mood Stabilizer | 14 (1.3%) | 3 (0.5%) | 17 (1.0%) |
| Nonsteroidal Anti-inflammatory Drug | 123 (11.3%) | 49 (7.7%) | 172 (10.0%) |
| Opioid | 40 (3.7%) | 32 (5.0%) | 72 (4.2%) |
| Selective Serotonin Reuptake Inhibitor | 137 (12.6%) | 53 (8.3%) | 190 (11.0%) |
| Serotonin and Norepinephrine Reuptake Inhibitor | 49 (4.5%) | 16 (2.5%) | 65 (3.8%) |
| Statin | 15 (1.4%) | 16 (2.5%) | 31 (1.8%) |
| Synthetic T4 | 24 (2.2%) | 7 (1.1%) | 31 (1.8%) |
| Tricyclic Antidepressant | 8 (0.7%) | 4 (0.6%) | 12 (0.7%) |

**Table S2. Participant Medication Use**. Medication classes were analyzed for subjects within two weeks prior to CRP testing. Several medications had the highest percentage of usage within overall subjects including SSRIs (11.0%), NSAIDs (10.0%), contraceptives (9.5%), antihistamines (8.8%), and non-SSRI anti-depressants (7.9%). Number in brackets represents percentage of population.

| **Table S3. Participant Medication Use Classified with Dichotomized CRP Levels** | | | |  | |
| --- | --- | --- | --- | --- | --- |
|  | **High CRP**  **lg CRP>0.02** | **Low CRP**  **lg CRP<0.02** | **Overall** | |  |
|  | **(N=860)** | **(N=864)** | **(N=1724)** | |  |
| Antibiotic | 24 (2.8%) | 28 (3.2%) | 52 (3.0%) | |  |
| Antidepressant(other) | 79 (9.2%) | 58 (6.7%) | 137 (7.9%) | |  |
| Antihistamine | 75 (8.7%) | 76 (8.8%) | 151 (8.8%) | |  |
| Antipsychotic | 22 (2.6%) | 12 (1.4%) | 34 (2.0%) | |  |
| Antiviral | 8 (0.9%) | 6 (0.7%) | 14 (0.8%) | |  |
| Benzodiazepine | 39 (4.5%) | 33 (3.8%) | 72 (4.2%) | |  |
| Central Nervous System Stimulant | 21 (2.4%) | 20 (2.3%) | 41 (2.4%) | |  |
| Contraceptive | 106 (12.3%) | 58 (6.7%) | 164 (9.5%) | |  |
| Mood Stabilizer | 13 (1.5%) | 4 (0.5%) | 17 (1.0%) | |  |
| Nonsteroidal Anti-inflammatory Drug | 104 (12.1%) | 68 (7.9%) | 172 (10.0%) | |  |
| Opioid | 53 (6.2%) | 19 (2.2%) | 72 (4.2%) | |  |
| Selective Serotonin Reuptake Inhibitor | 107 (12.4%) | 83 (9.6%) | 190 (11.0%) | |  |
| Serotonin and Norepinephrine Reuptake Inhibitor | 35 (4.1%) | 30 (3.5%) | 65 (3.8%) | |  |
| Statin | 21 (2.4%) | 10 (1.2%) | 31 (1.8%) | |  |
| Synthetic T4 | 19 (2.2%) | 12 (1.4%) | 31 (1.8%) | |  |
| Tricyclic Antidepressant | 6 (0.7%) | 6 (0.7%) | 12 (0.7%) | |  |

**Table S3. Participant Medication Use Classified by Dichotomized CRP Levels.** Several medications had a significantly higher use in the high versus the low CRP group as indicated by Chi-squared test: contraceptive (*Χ*^2^= 0.158; p= 7.14E-05), mood stabilizer (*Χ*^2^= 4.85; p= 0.028), NSAID (*Χ*^2^= 8.56; p= 0.003), opioid (*Χ*^2^= 0.169; p= 3.90E-05), and statin (*Χ*^2^= 4.03; p= 0.045).

**Table S4. Durbin Watson Test for Autocorrelation**

|  |  |  |  |
| --- | --- | --- | --- |
|  | Autocorrelation | D-W Statistic | p-value |
| All | -0.186 | 2.036 | 0.468 |
| Female | -0.473 | 2.093 | 0.148 |
| Male | 0.006 | 1.987 | 0.824 |

**Table S4. Durbin Watson Test for Autocorrelation.** The Durbin Watson Test was used to test whether data were autocorrelated. Our datasets are not autocorrelated, which was determined by the following p-values: All (p= 0.468), Female (p= 0.148), and Male (p= 0.824).

**Table S5. Variance Inflation Factor (VIF) for Multicollinearity.**

|  | All | Female | Male |
| --- | --- | --- | --- |
| Age | 1.379 | 1.363 | 1.559 |
| Gender | 3.054 |  |  |
| Hispanic/Latino | 1.200 | 1.240 | 1.186 |
| White | 2.545 | 2.421 | 3.067 |
| African American | 2.157 | 2.124 | 2.438 |
| Native American | 1.294 | 1.304 | 1.329 |
| Asian | 1.130 | 1.113 | 1.214 |
| Other | 1.502 | 1.468 | 1.700 |
| Income | 1.252 | 1.317 | 1.264 |
| Education | 1.355 | 1.405 | 1.351 |
| Alcohol Use | 1.105 | 1.116 | 1.113 |
| Caffeine Use | 1.178 | 1.152 | 1.302 |
| Nicotine Use | 1.484 | 1.401 | 1.605 |
| Body Mass Index | 4.052 | 4.272 | 3.992 |
| Percent Body Fat | 5.758 | 4.144 | 4.442 |
| Waist to hip Ratio | 1.782 | 1.450 | 1.919 |
| Patient Health Questionnaire Score | 1.442 | 1.445 | 1.537 |
| Drug Use Questionnaire Score | 1.600 | 1.591 | 1.652 |
| QIDS-SR Sleep Score | 1.435 | 1.424 | 1.546 |
| C-reactive Protein Detection Range | 1.068 | 1.091 | 1.082 |
| Antibiotic | 1.045 | 1.063 | 1.050 |
| Antidepressant(other) | 1.187 | 1.187 | 1.310 |
| Antihistamine | 1.099 | 1.116 | 1.109 |
| Antipsychotic | 1.111 | 1.115 | 1.201 |
| Antiviral | 1.023 | 1.044 | 1.048 |
| Benzodiazepine | 1.124 | 1.152 | 1.183 |
| Central Nervous System Stimulant | 1.035 | 1.066 | 1.023 |
| Contraceptive |  | 1.118 |  |
| Mood Stabilizer | 1.062 | 1.103 | 1.077 |
| Nonsteroidal Anti-inflammatory Drug | 1.060 | 1.071 | 1.096 |
| Opioid | 1.076 | 1.125 | 1.070 |
| Selective Serotonin Reuptake Inhibitor | 1.146 | 1.182 | 1.197 |
| Serotonin and Norepinephrine Reuptake Inhibitor | 1.097 | 1.116 | 1.146 |
| Statin | 1.079 | 1.083 | 1.152 |
| Synthetic T4 | 1.047 | 1.066 | 1.053 |
| Tricyclic Antidepressant | 1.033 | 1.049 | 1.074 |

**Table S5. Variance Inflation Factor (VIF) for Multicollinearity.** VIF was used to check the amount of multicollinearity in all datasets. Body mass index and percent body fat have the highest VIF across all datasets.

| **Table S6. Individual PHQ-9 Items and Association with CRP** | | |  |
| --- | --- | --- | --- |
|  | **Female** | **Male** | **Overall** |
|  | **(N=1086)** | **(N=638)** | **(N=1724)** |
| **Patient Health Questionnaire Score Q1 (anhedonia)** |  |  |  |
| Mean (SD) | 1.05 (1.00) | 1.07 (1.02) | 1.06 (1.01) |
| Median [Min, Max] | 1.00 [0, 3.00] | 1.00 [0, 3.00] | 1.00 [0, 3.00] |
| **Patient Health Questionnaire Score Q2 (depressed)** |  |  |  |
| Mean (SD) | 1.14 (1.04) | 1.04 (1.01) | 1.10 (1.03) |
| Median [Min, Max] | 1.00 [0, 3.00] | 1.00 [0, 3.00] | 1.00 [0, 3.00] |
| **Patient Health Questionnaire Score Q3 (sleep)** |  |  |  |
| Mean (SD) | 1.52 (1.17) | 1.41 (1.19) | 1.48 (1.18) |
| Median [Min, Max] | 1.00 [0, 3.00] | 1.00 [0, 3.00] | 1.00 [0, 3.00] |
| **Patient Health Questionnaire Score Q4 (tired)** |  |  |  |
| Mean (SD) | 1.62 (1.09) | 1.39 (1.09) | 1.53 (1.09) |
| Median [Min, Max] | 2.00 [0, 3.00] | 1.00 [0, 3.00] | 1.00 [0, 3.00] |
| **Patient Health Questionnaire Score Q5 (appetite)** |  |  |  |
| Mean (SD) | 1.13 (1.10) | 0.939 (1.01) | 1.06 (1.07) |
| Median [Min, Max] | 1.00 [0, 3.00] | 1.00 [0, 3.00] | 1.00 [0, 3.00] |
| **Patient Health Questionnaire Score Q6 (feeling like a failure)** |  |  |  |
| Mean (SD) | 1.25 (1.12) | 1.23 (1.12) | 1.24 (1.12) |
| Median [Min, Max] | 1.00 [0, 3.00] | 1.00 [0, 3.00] | 1.00 [0, 3.00] |
| **Patient Health Questionnaire Score Q7 (trouble concentrating)** |  |  |  |
| Mean (SD) | 1.03 (1.06) | 1.04 (1.06) | 1.03 (1.06) |
| Median [Min, Max] | 1.00 [0, 3.00] | 1.00 [0, 3.00] | 1.00 [0, 3.00] |
| **Patient Health Questionnaire Score Q8 (slow/fidgety/restless)** |  |  |  |
| Mean (SD) | 0.555 (0.864) | 0.663 (0.936) | 0.595 (0.893) |
| Median [Min, Max] | 0 [0, 3.00] | 0 [0, 3.00] | 0 [0, 3.00] |
| **Patient Health Questionnaire Score Q9 (suicidal)** |  |  |  |
| Mean (SD) | 0.276 (0.616) | 0.293 (0.654) | 0.282 (0.630) |
| Median [Min, Max] | 0 [0, 3.00] | 0 [0, 3.00] | 0 [0, 3.00] |

**Table S6.** **Individual Items from the PHQ-9.** Our analysis showed that for the overall sample (β: min: -0.03; max: 0.02) and male-only sample (β: min: -0.05; max: 0.04) there were no significant relationships between CRP and any of the individual PHQ-9 items in the fully adjusted model (p-values>0.20; results not shown). For females, only item 3 (trouble falling or staying asleep, or sleeping too much) and item 9 (thoughts that you would be better off dead, or of hurting yourself) showed marginal significance (β= -0.059; p=0.054) and (β= -0.053; p= 0.04), respectively.

**Table S7. Standardized Beta Coefficients and Pearson's Correlations for PHQ-9 Total Score vs. PHQ-9 (no sleep item)**

|  | Standardized Beta (β) | Pearson's Correlation (*r*) |
| --- | --- | --- |
| PHQ-9 | All (β= -4.0E-3)  Female (β= -3.4 E-2)  Male (β= 9.2E-3) | All (*r*= 0.13)  Female (*r*= 0.09)  Male (*r*= 0.18) |
| PHQ-9  (No sleep item) | All (β= 9.0E-4)  Female (β= -2.7E-2)  Male (β= 1.0E-2) | All (*r*= 0.12)  Female (*r*= 0.09)  Male (*r*= 0.17) |

**Table S7. Standardized Beta Coefficients and Pearson's Correlations for PHQ-9 Total Score vs. PHQ-9 (no sleep item).** Standardized beta coefficients and correlations for PHQ-9 total score compared to PHQ-9 (no sleep item) were not different.

**Figure S1. CONSORT Flow Diagram**

**
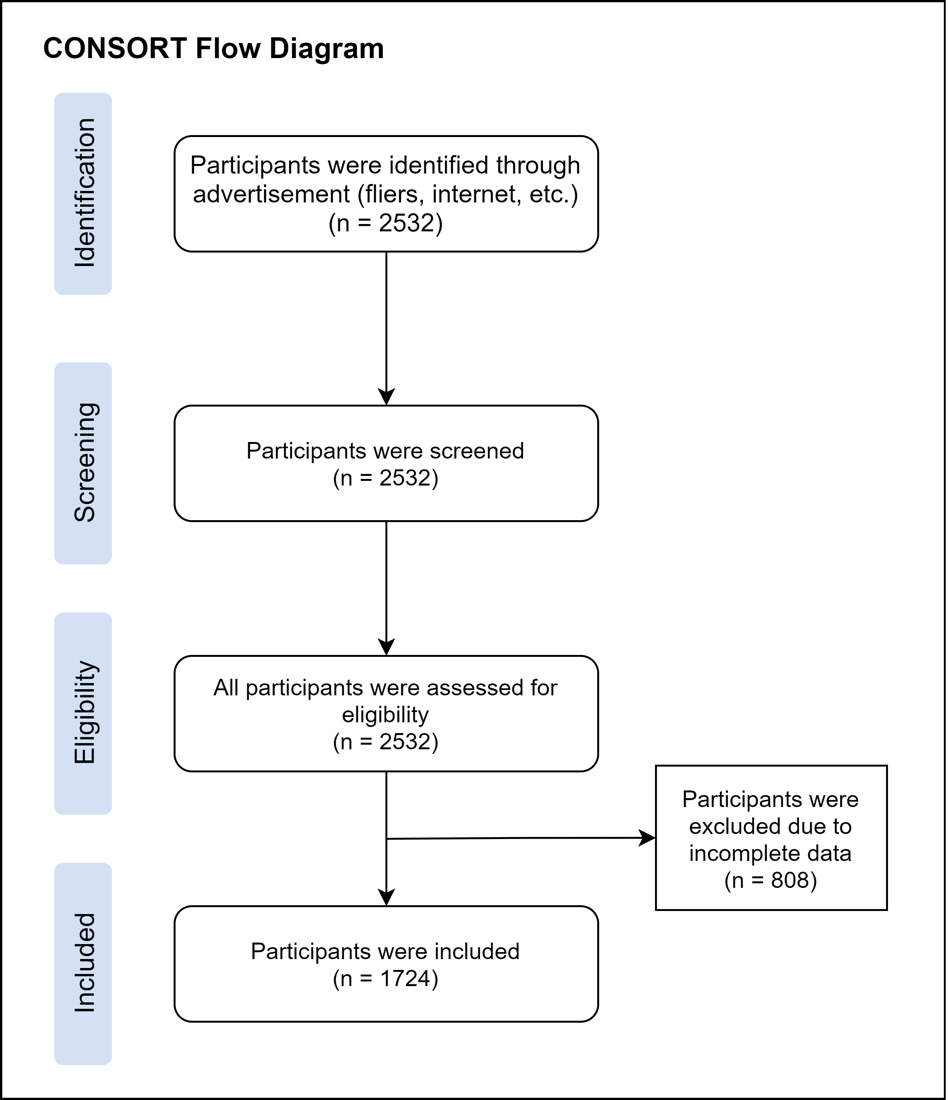
**

**Figure S1. CONSORT Flow Diagram.** The CONSORT flow diagram shows identification**,** screening, and eligibility for all participants (n=2532), participants that were excluded for incomplete data (n=808), and participants that were included in the multivariate linear analysis of the main manuscript (n=1724).

**Figure S2. Aggregation Plot for Missing Data Variables**

**
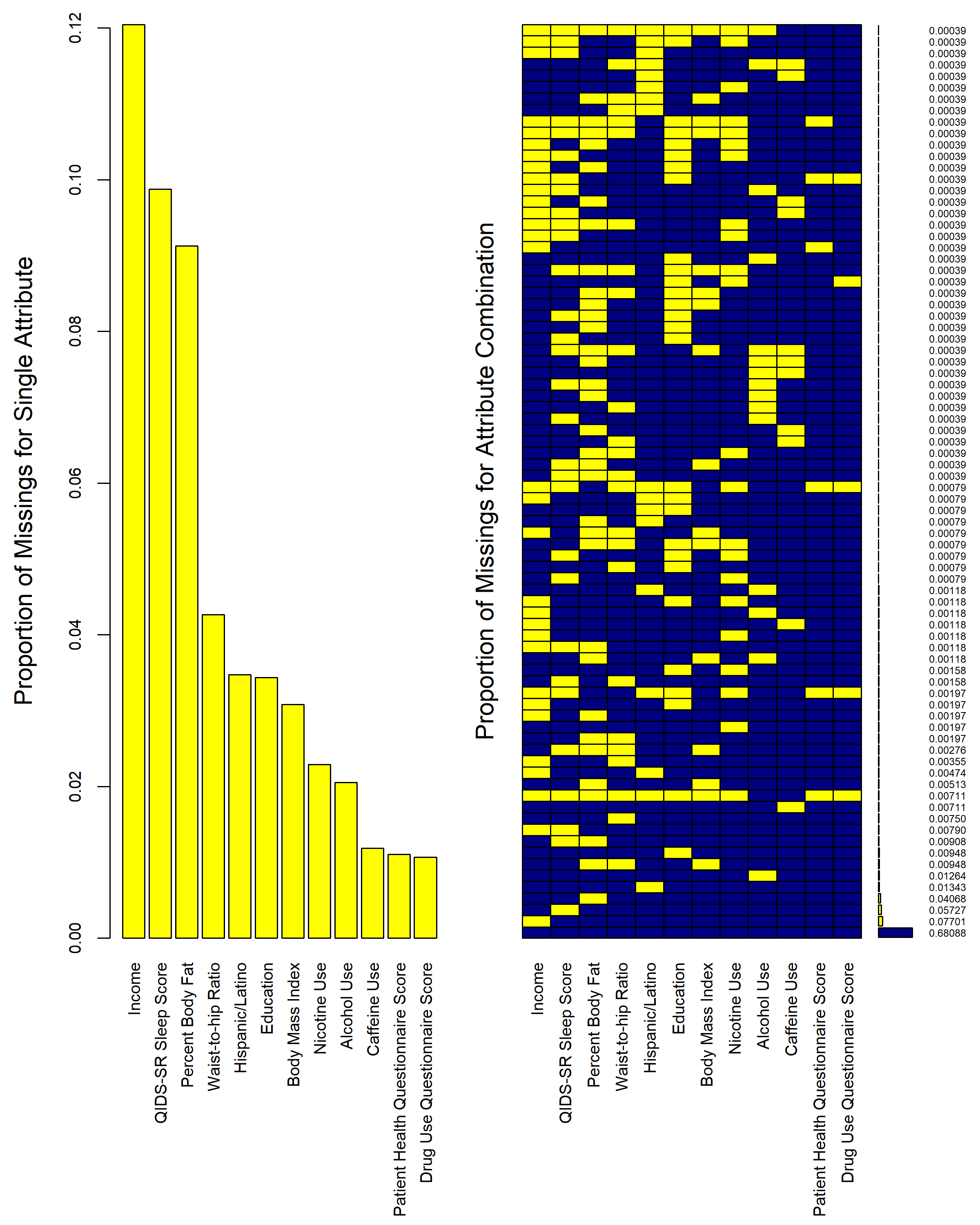
**

**Figure S2. Aggregation Plot for Missing Data Variables**. The aggregation plot was used to visualize the missing data from 808 subjects. Annual family income had the most missing values.

**Figure S3. C-reactive Protein Distribution**

1. **B)**


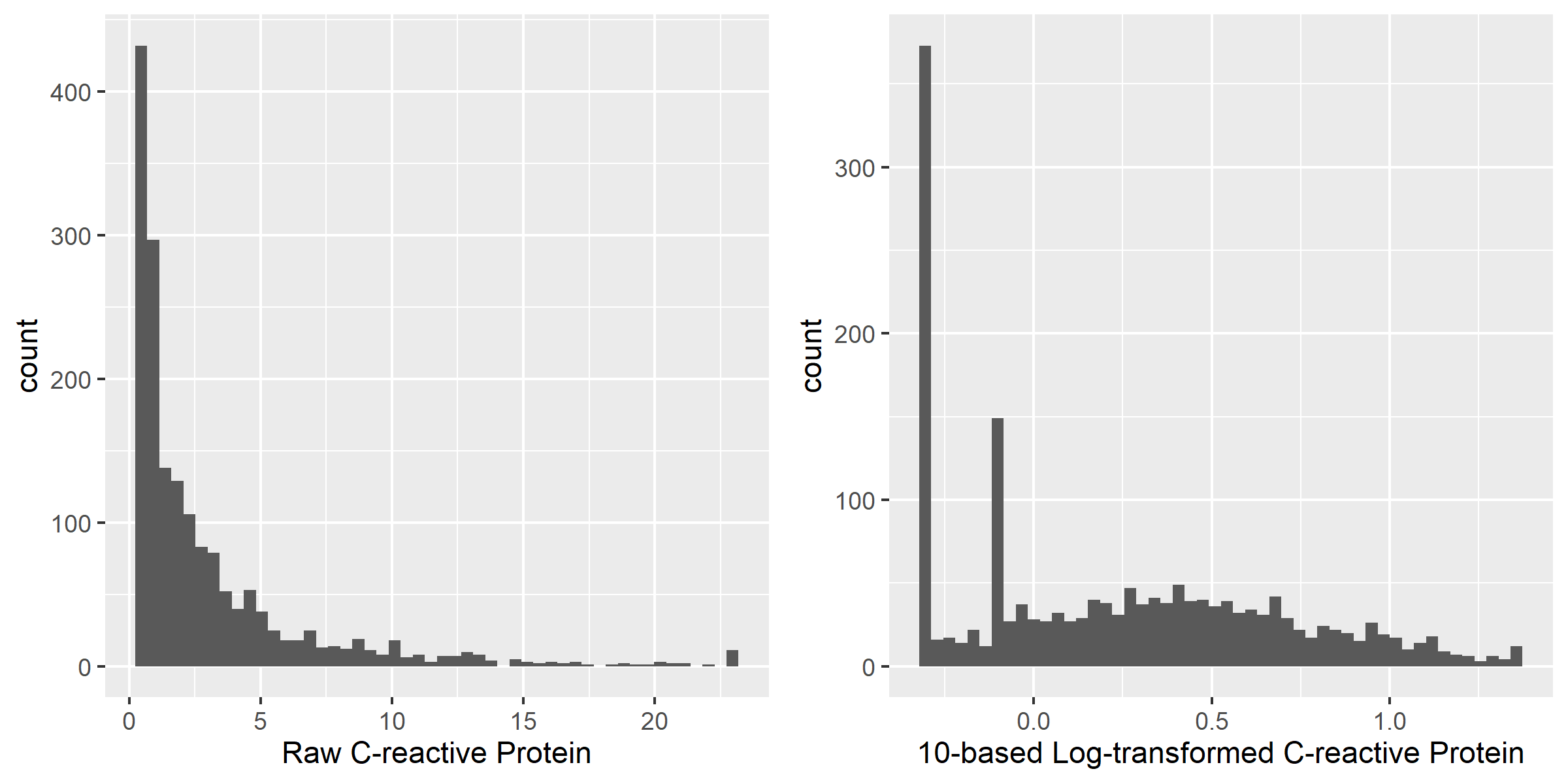


**Figure S3. C-reactive Protein Distribution.** Raw and log-transformed C-reactive Protein (CRP) levels were plotted to visualize CRP distribution. **A)** Distribution of raw CRP levels for our study population and **B)** distribution of log-transformed CRP levels. The slight difference in detection ranges (0.5-23) and (0.8-20) resulted in two taller bars, which represent subjects whose CRP levels below the lower limits of detection.

**Figure S4. C-reactive Protein Daily Pattern.**

1. All Subjects (2532)


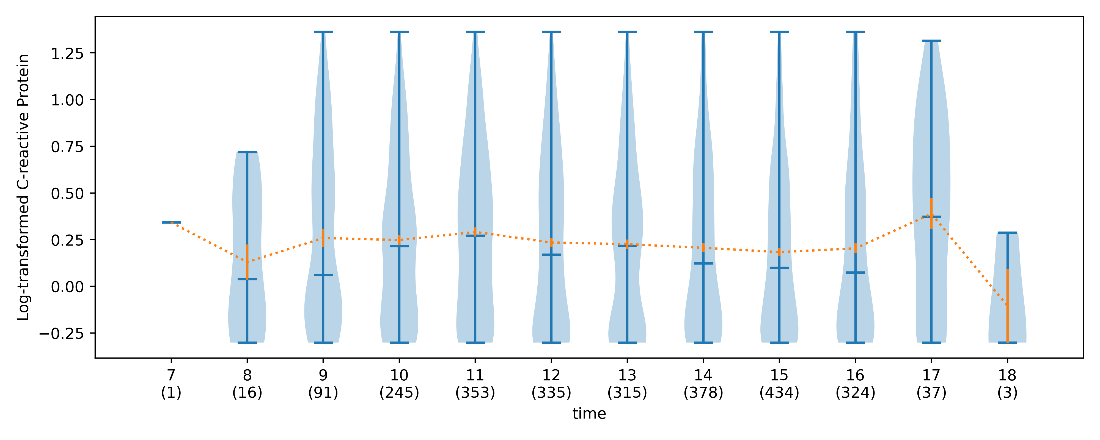


1. Female Subjects (1594)


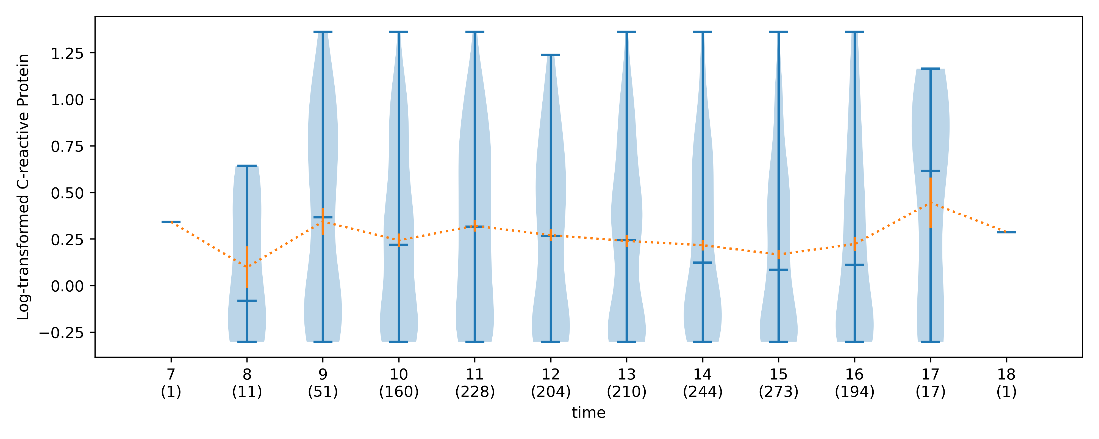


1. Male Subjects (938)


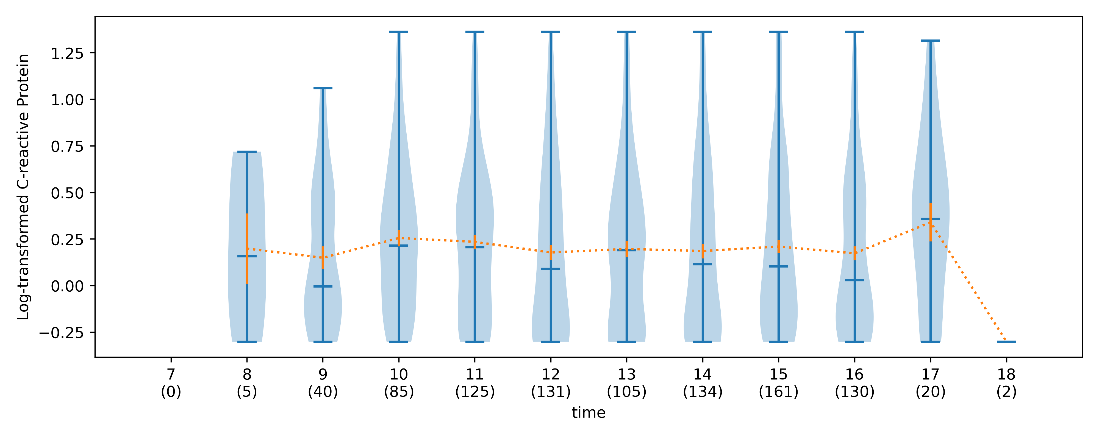


**Figure S4. C-reactive Protein Daily Pattern.** The CRP daily pattern was analyzed from 7 am to 6 pm. **A)** CRP daily pattern for all subjects (n=2532), **B)** females (n=1594), and **C)** males (n=938). Results reported as log-transformed CRP. Number in parenthesis denote number of participants tested during that time point. The violin plots represent maximum, median, and minimum log-transformed CRP levels (blue bars), respectively. The mean and corresponding standard error is represented by the orange dash line and error bars, respectively. Values for A) all subjects: 0.006, B) females: 0.015, and C) males: 0.018, indicate almost no mutual dependence between log-transformed CRP and measurement time.

**Figure S5. C-reactive Protein Yearly Pattern**

1. All Subjects (2532)


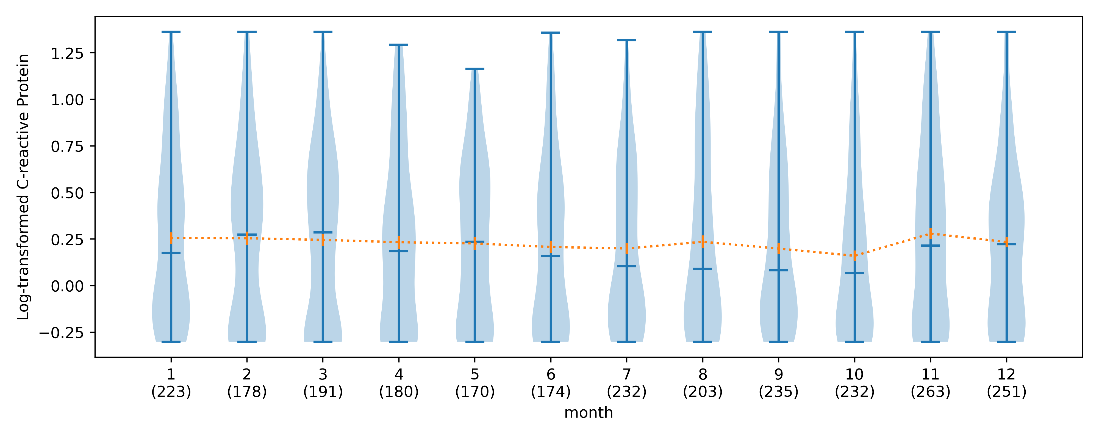


1. Female Subjects (1594)


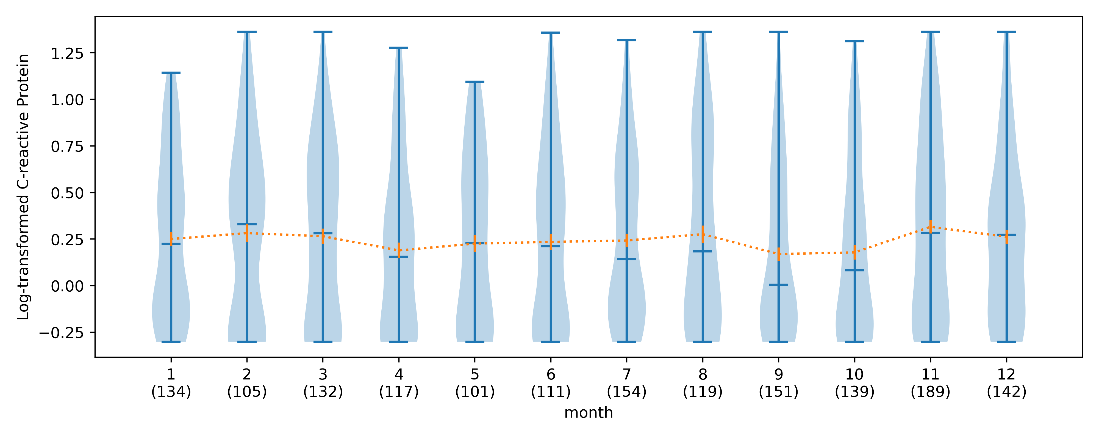


1. Male Subjects (938)


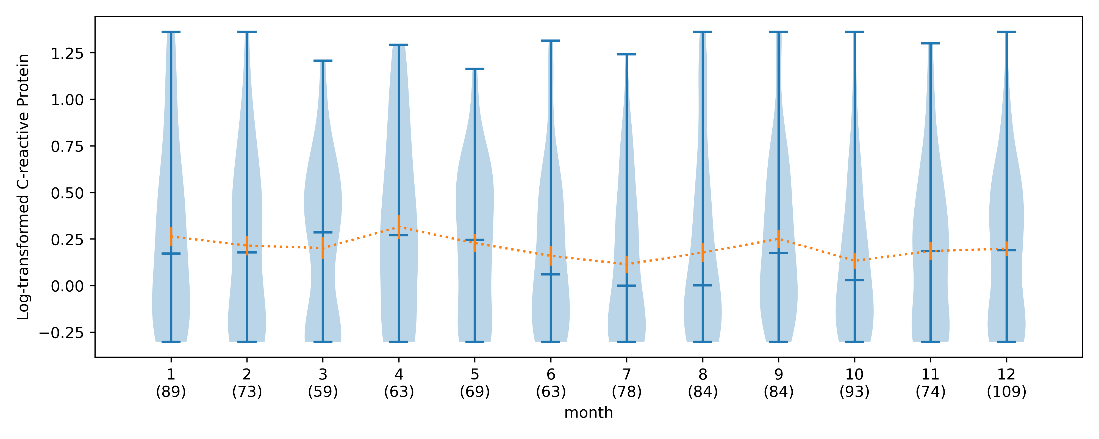


**Figure S5. C-reactive Protein Yearly Pattern.** The CRP yearly pattern was analyzed each month over 12 months. **A)** CRP yearly pattern for all subjects (n=2532), **B)** females (n=1594), **C)** males (n=938). Values for A) all subjects: 0.051, B) females: 0.049, and C) males: 0.004, indicate almost no mutual dependence between log-transformed CRP and measurement time.

**Figure S6. Residual vs. Fitted Plots**

**
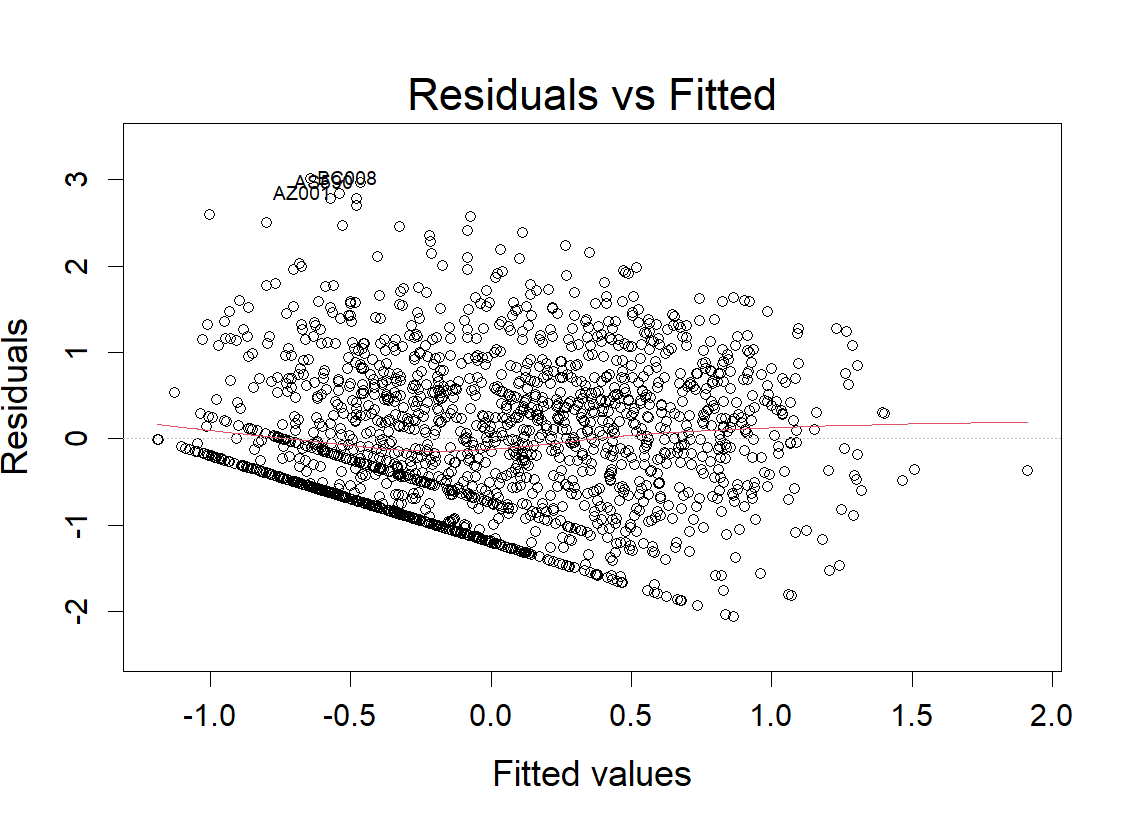
**

**Figure S6A.** Testing the linearity of the data for all subjects.

**
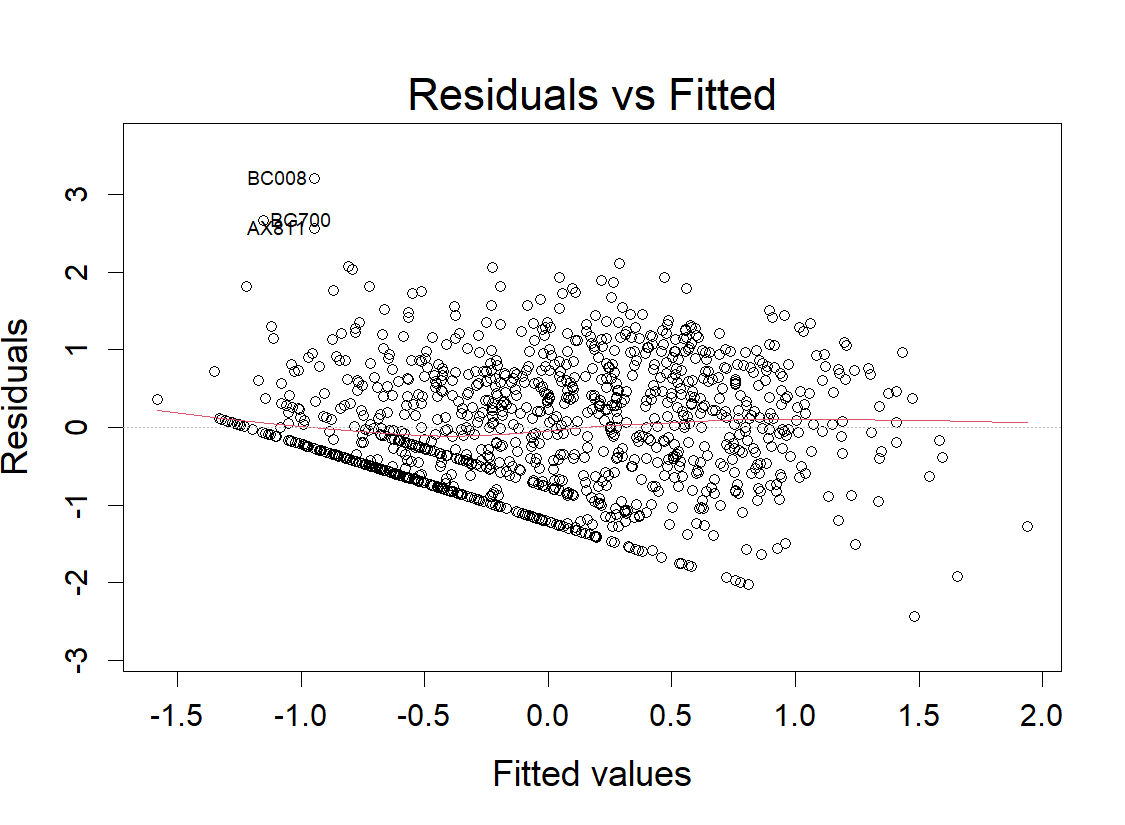
**

**Figure S6B.** Testing the linearity of the data for female subjects.

**
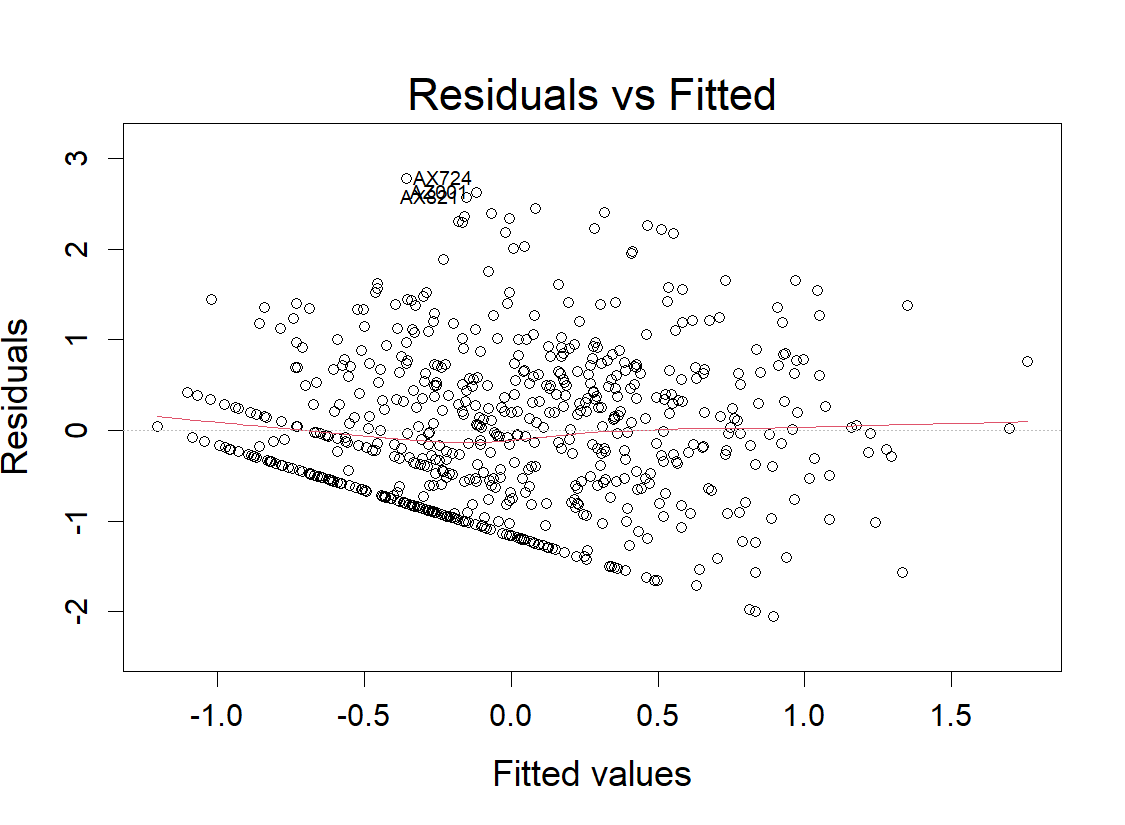
**

**Figure S6C.** Testing the linearity of the data male subjects.

**Figure S6. Residual vs. Fitted Plots.** Testing the linearity of the data for **A)** all subjects, **B)** females, and **C)** males. Two data point lines in all plots indicate limit of detection from 2 peaks in log transformed CRP distribution in Figure S3. Flat red curves for all plots indicate linear assumption is valid on this data for linear model.

**Figure S7. Quartile-Quartile Plots**

**
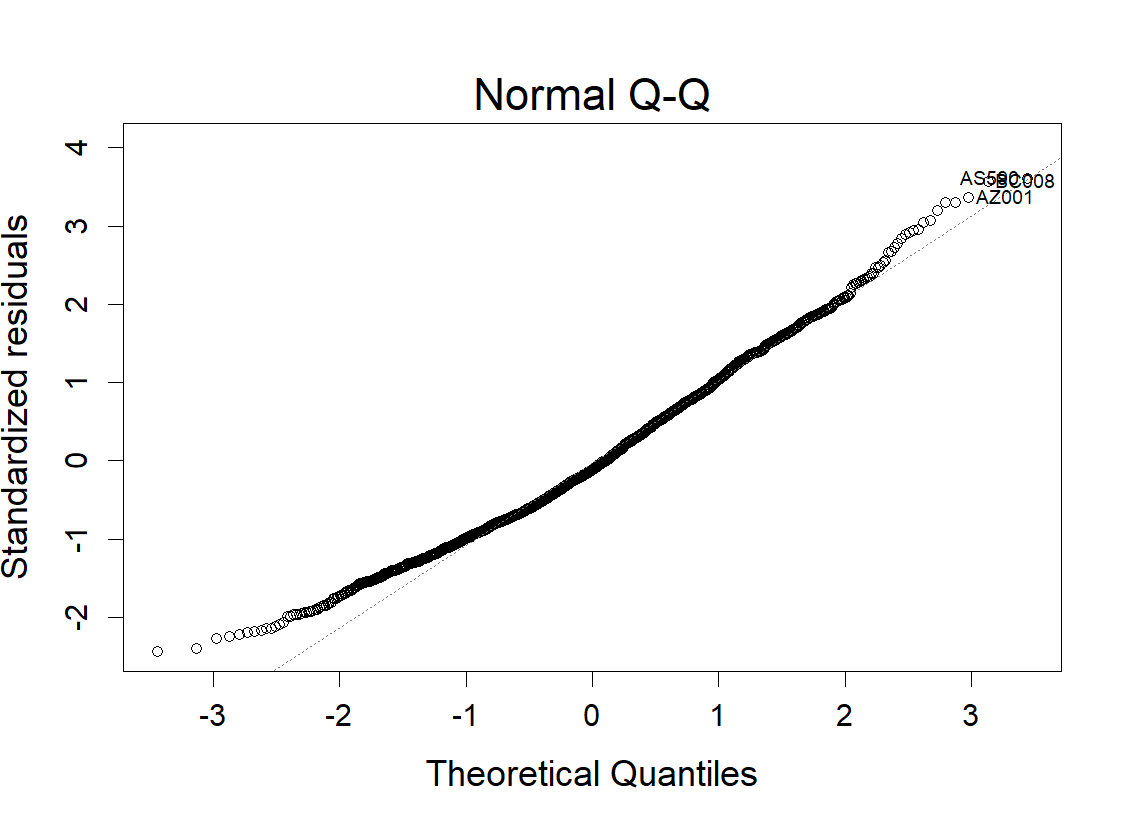
**

**Figure S7A.** QQ plots for all subjects.

**
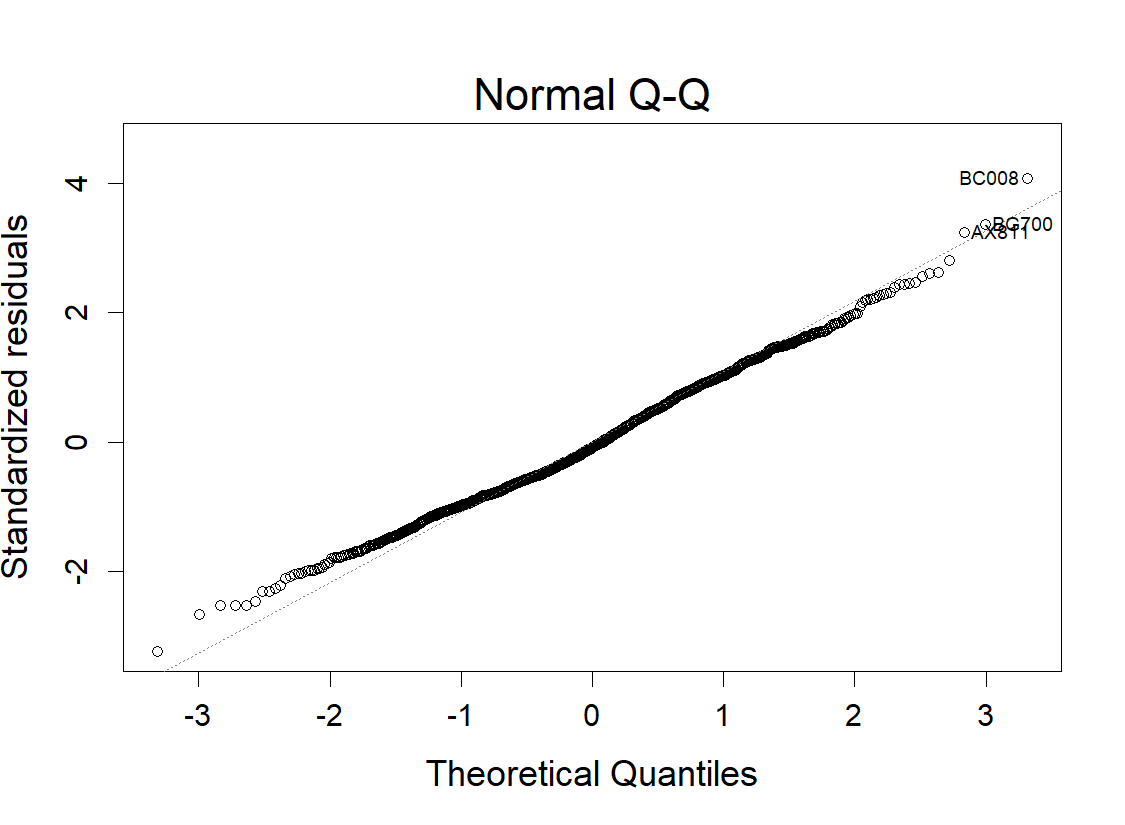
**

**Figure S7B.** QQ plots for female subjects.


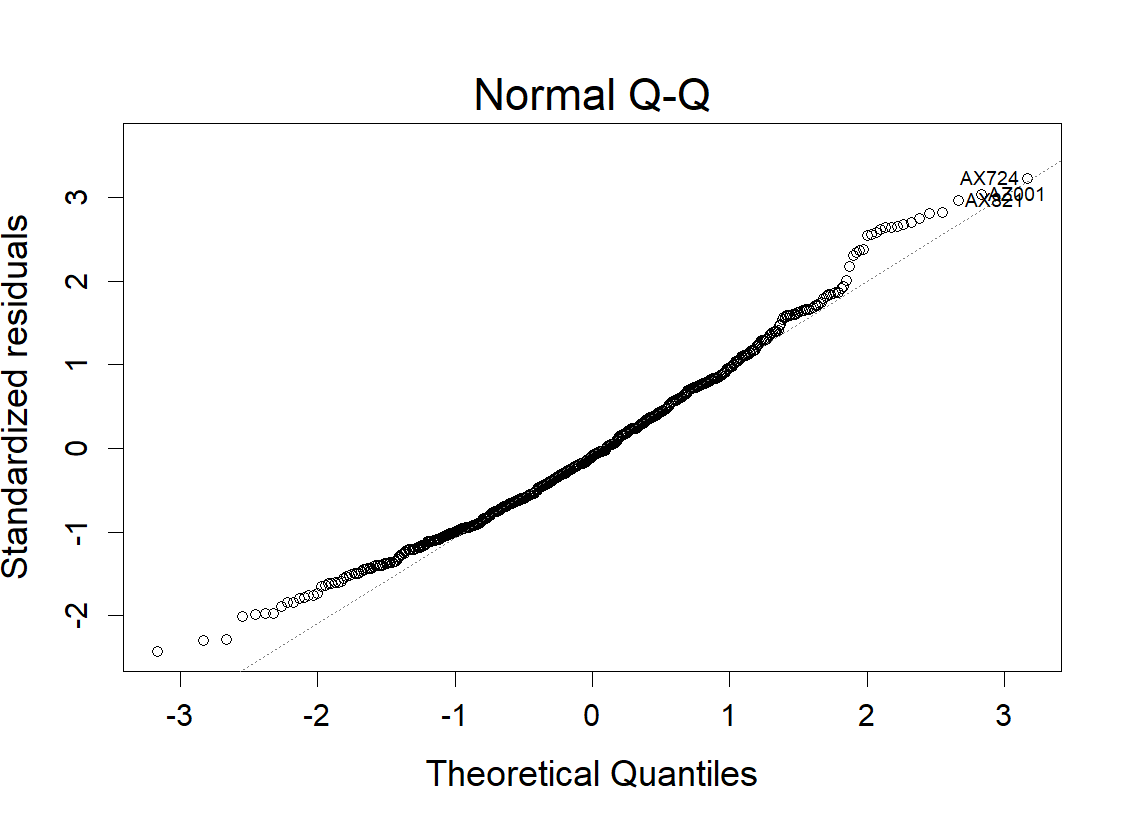


**Figure S7C.** QQ plots for male subjects.

**Figure S7. Quartile-Quartile (Q-Q) Plots.** Testing the normality of the residuals for **A)** all subjects, **B)** females, and **C)** males. These plots show residuals are normally distributed.

**Figure S8. Residual vs Leverage Plots**

**
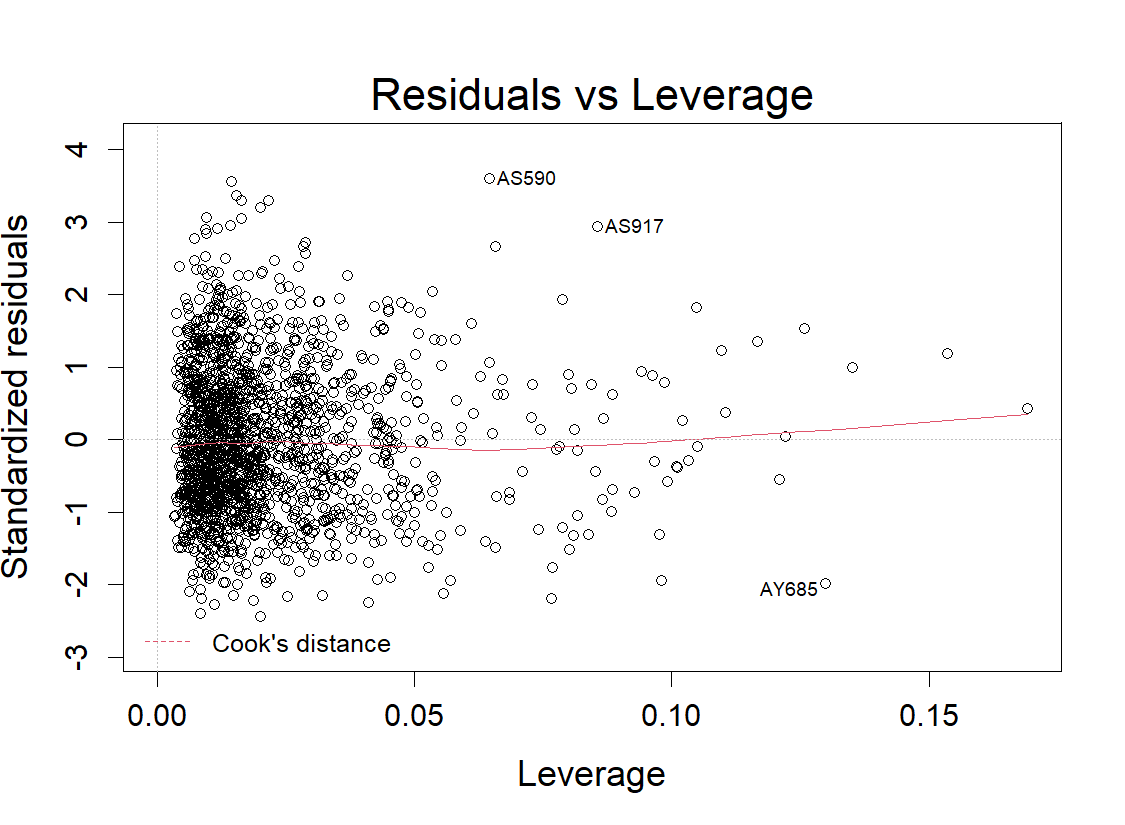
**

**Figure S8A.** Influence of outliers for all subjects.

**
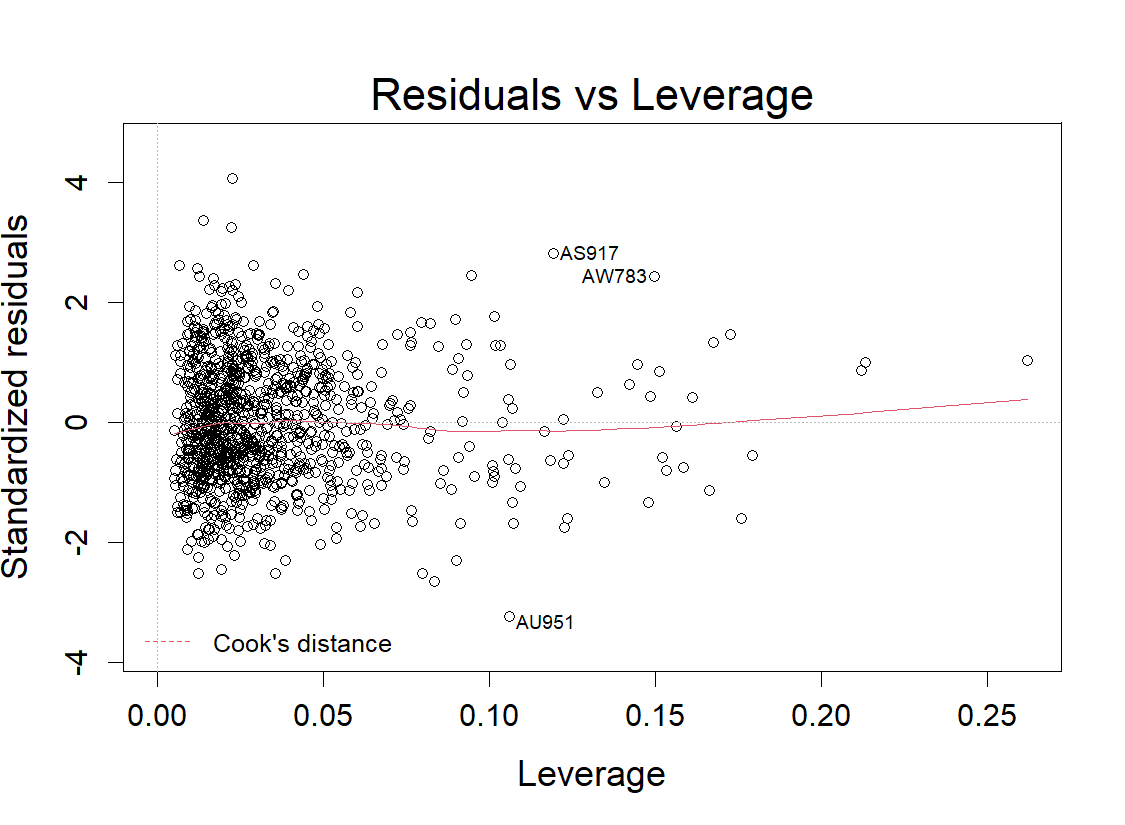
**

**Figure S8B.** Influence of outliers for female subjects.


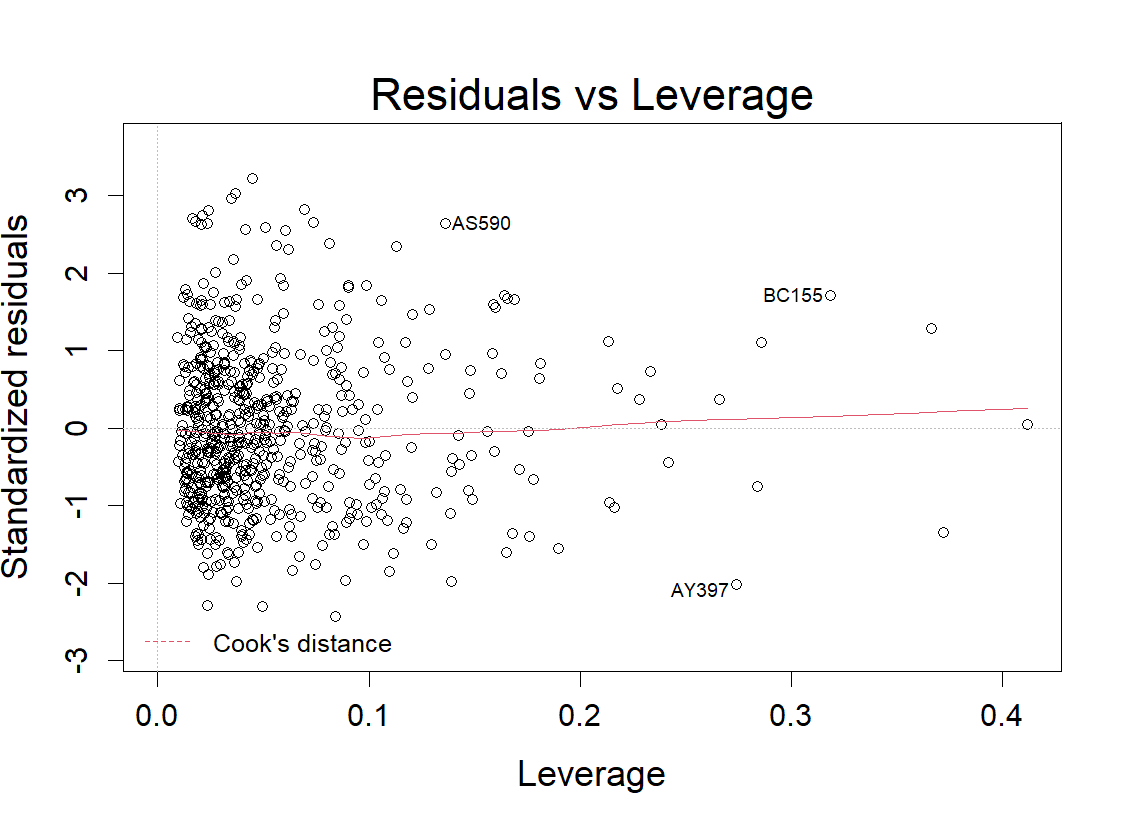


**Figure S8C.** Influence of outliers for male subjects.

**Figure S8. Residual vs. Leverage Plots.** These plots were used to identify any outliers, which would potentially have significant effect on the model fitting. Plots for **A)** all subjects, **B)** females, and **C)** males.

**Supplemental analyses with depressed group only (PHQ-9 score of greater than or equal to 9; n= 787); Figures S9-S12**

**Figure S9. Association between CRP and Total PHQ-9**


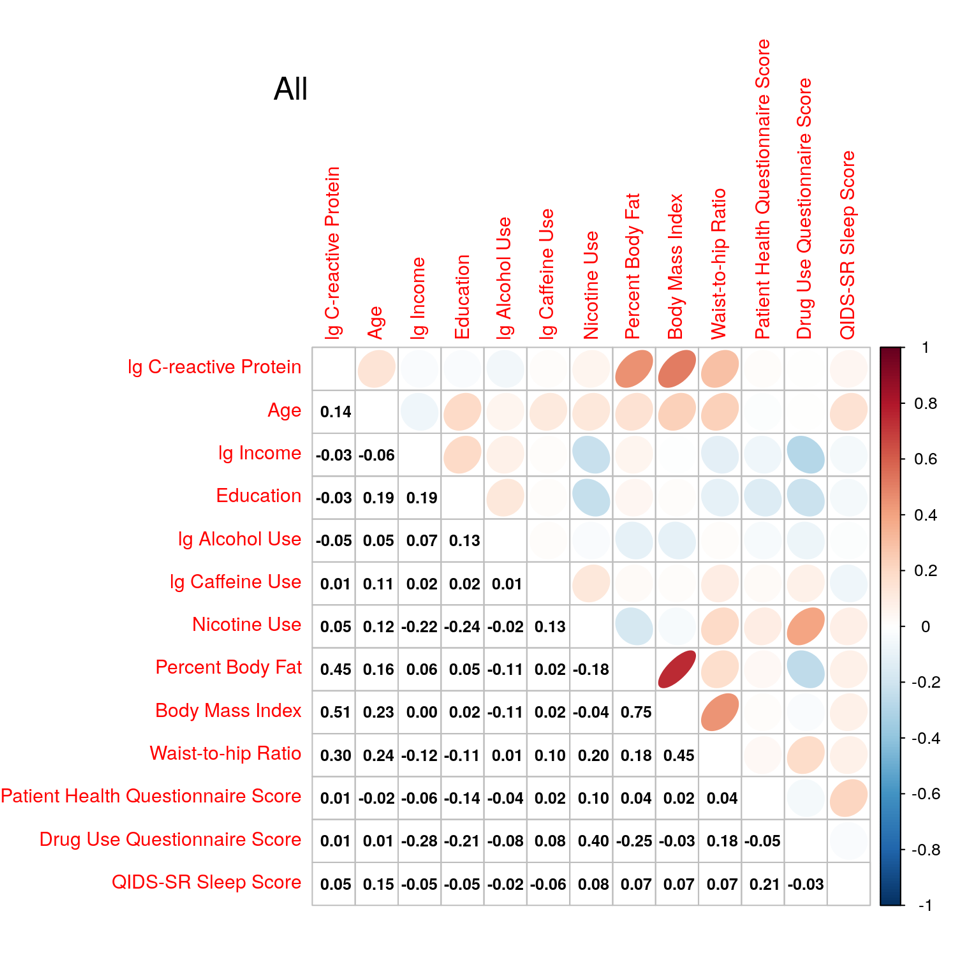


**Figure S9. Association between CRP and Total PHQ-9.** Correlations between CRP and PHQ-9 did not improve; including all participants led to a correlation of (*r*= 0.13) and depressed only (*r*= 0.01).

**Figure S10. Association between CRP and Individual Symptoms (PHQ-9 individual items)**


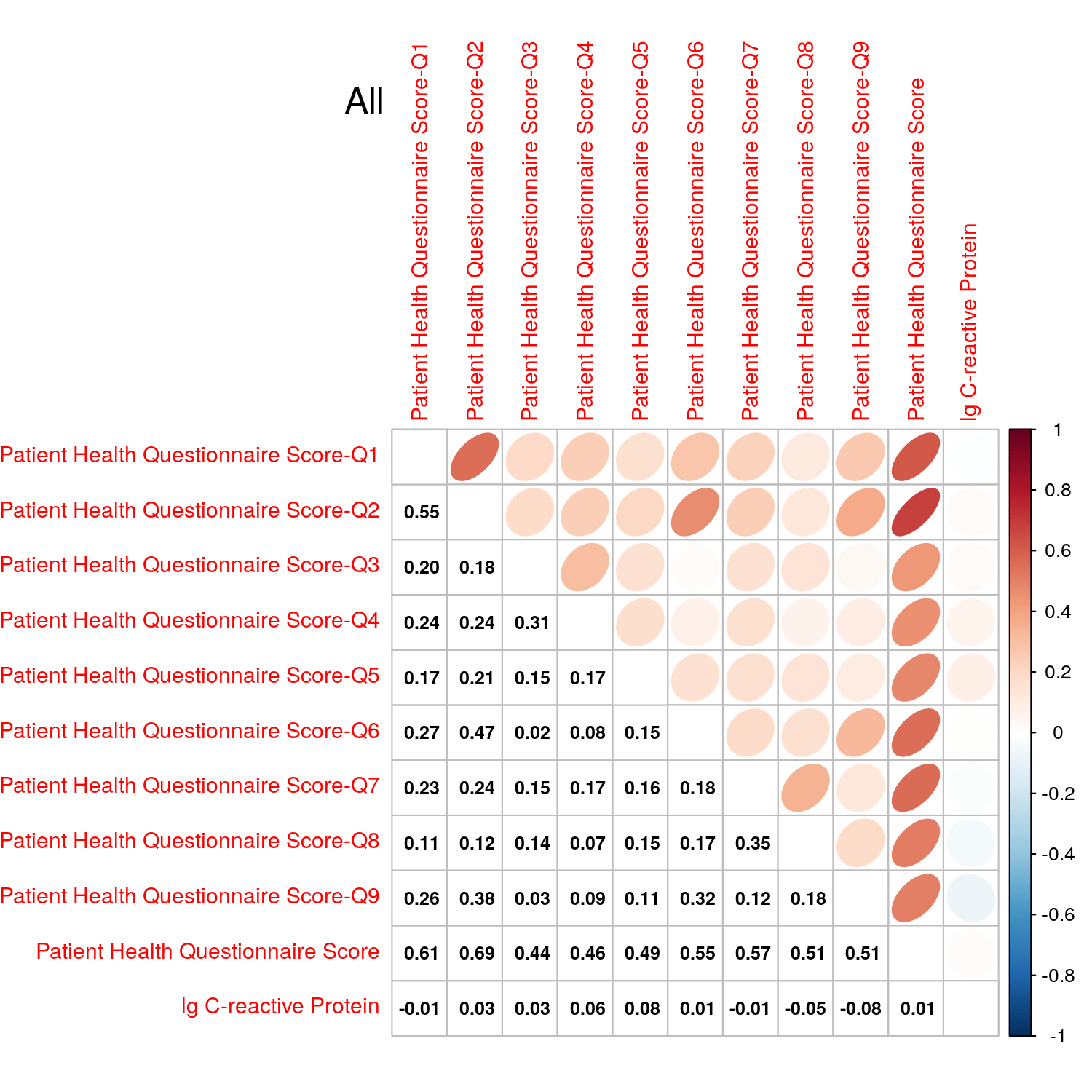


**Figure S10. Association between CRP and Individual Symptoms (PHQ-9 individual items).** Individual symptom items were highly correlated with each other in general. The range of Pearson correlation coefficients between CRP and PHQ individual symptom/total score ranges from -0.08 to 0.08.

**Figure S11. Standardized Beta Coefficients of CRP and all Covariates**


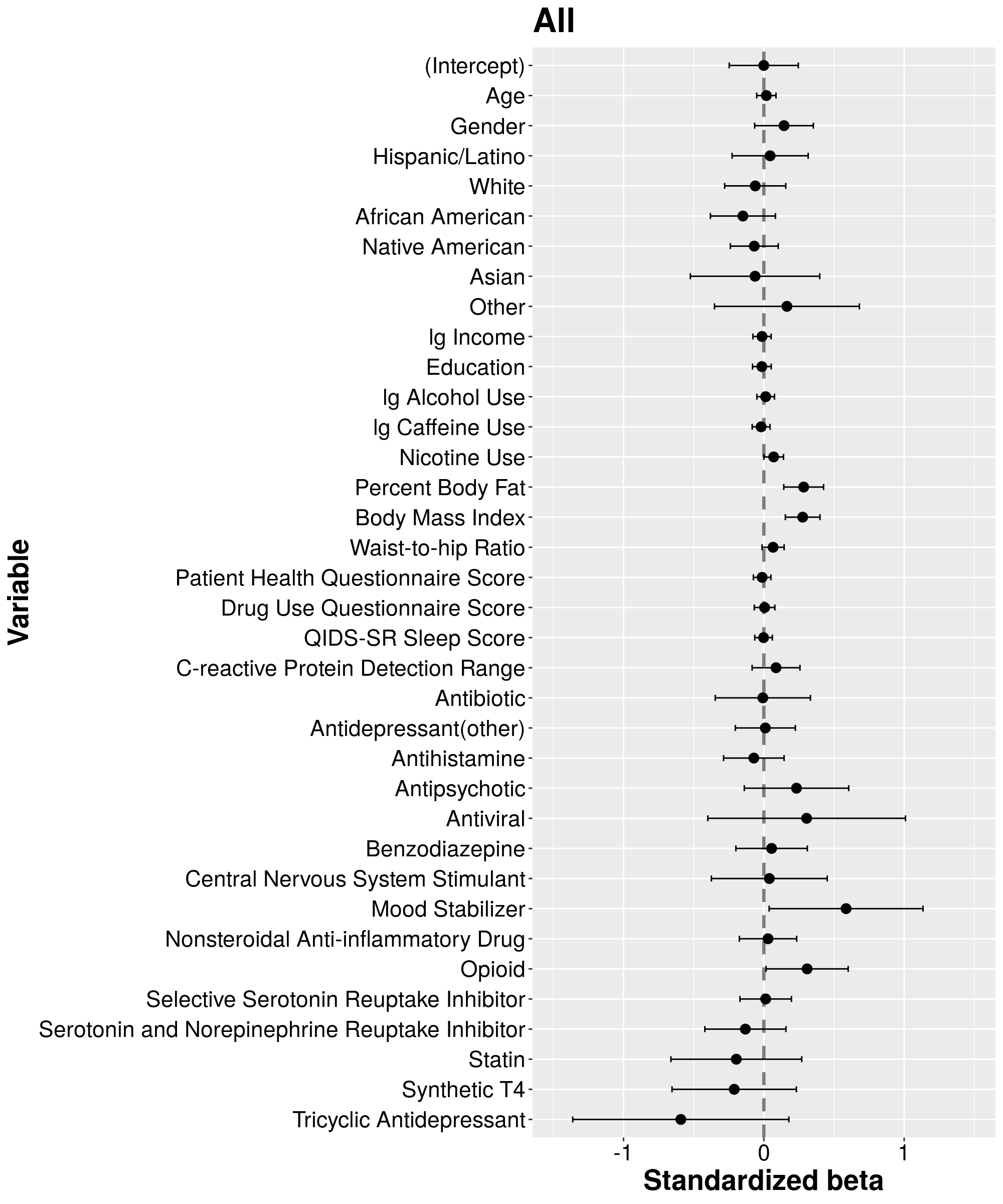


**Figure S11. Standardized Beta Coefficients of CRP and all Covariates.** Standardized beta coefficients show that CRP was significantly associated with nicotine use (β = 0.07, p=0.05), PBF (β = 0.284, p=9.38E-5), BMI (β = 0.277, p=1.26E-5), mood stabilizer use (β = 0.586, p=0.036), and opioid use (β = 0.308, p=0.039).

**Figure S12. Standardized Beta Coefficients for Main Variables Impacting PHQ-9 and CRP**


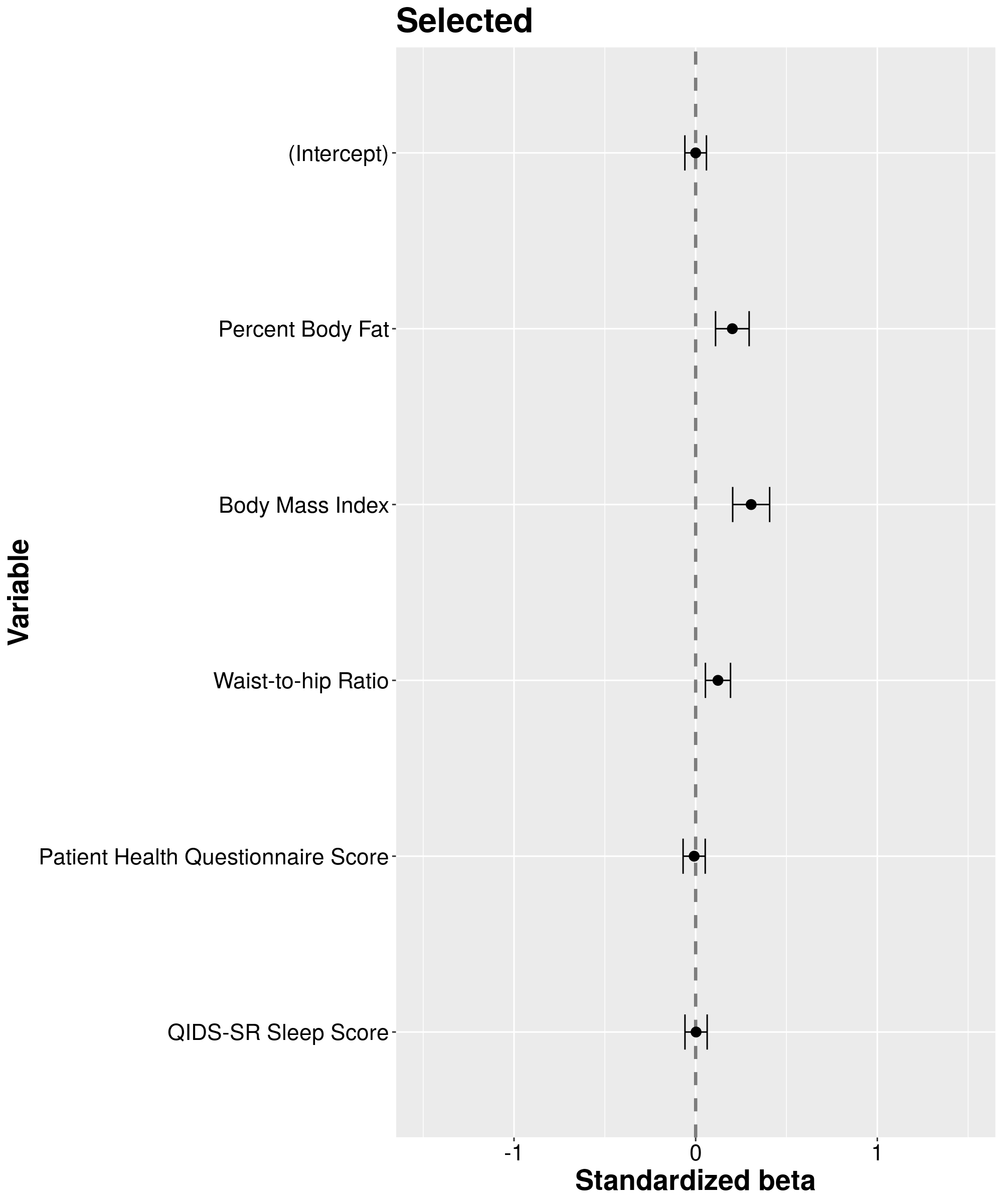


**Figure S12. Standardized Beta Coefficients for Main Variables Impacting PHQ-9 and CRP.** Standardized beta coefficients show that PBF (β = 0.202, p=2.04E-5), BMI (β = 0.306, p=5.55E-9), and WHR (β = 0.123, p=0.001), were all significantly associated with CRP when looking at depressed individuals with PHQ-9 of equal to or greater than a score of 10.

**Machine Learning Analysis (n=1724)**

**Figure S13. Nested Cross Validation**


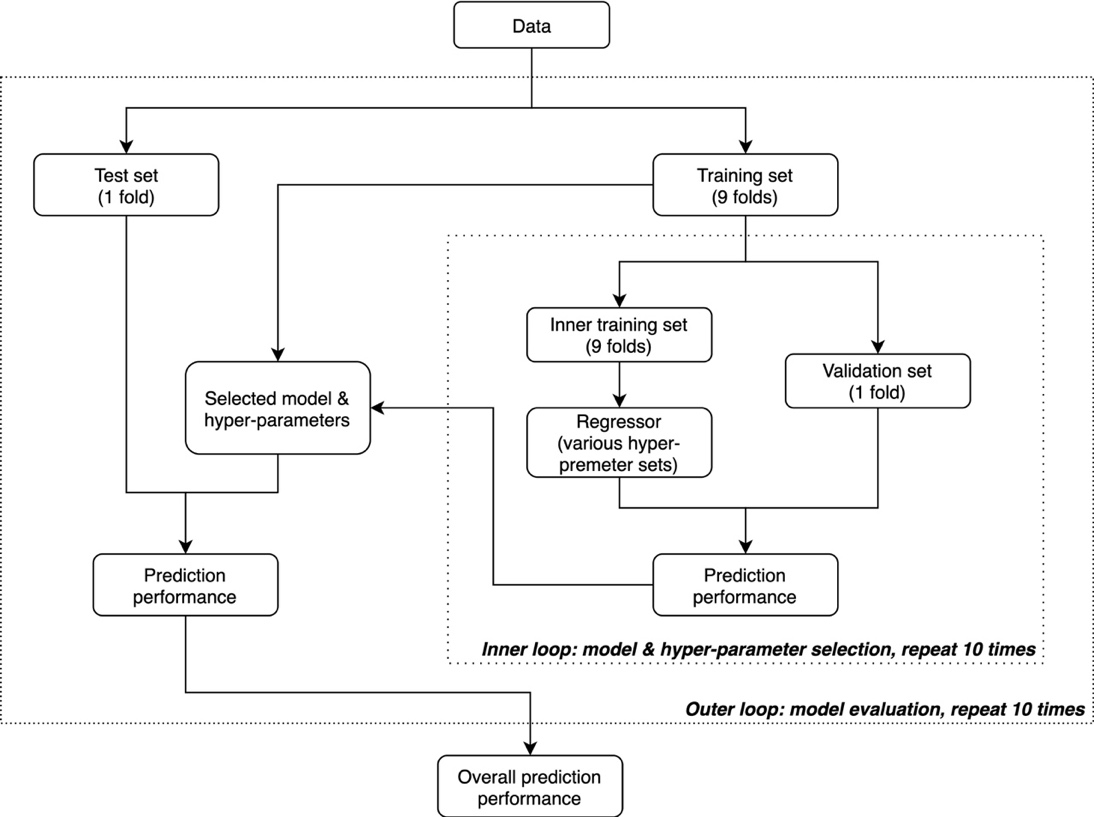


**Figure S13. Nested Cross-Validation.** This procedure was used to search for optimal model hyper-parameters and obtain a robust and unbiased evaluation of model performance**.**

**Figure S14. R2 Values**

(A) All Subjects (B) Female Subjects (C) Male Subjects


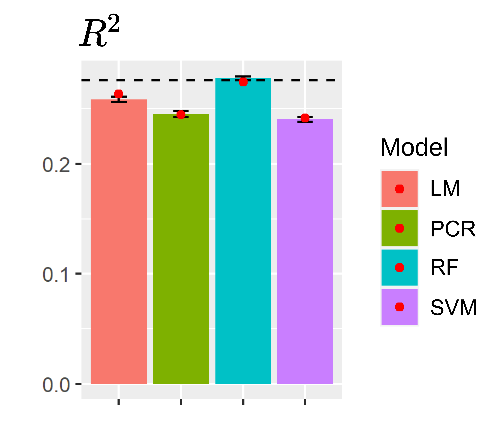

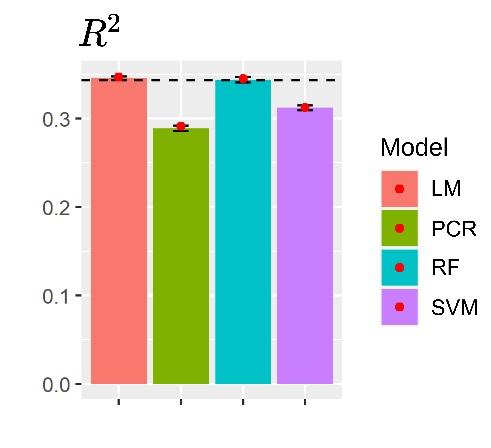

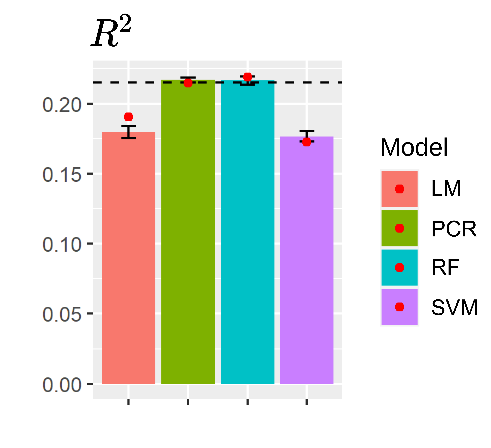


**Figure S14. Multivariate linear regression, Principal Component Regression, Random Forest, and Support Vector Machine Models.** Multivariate linear regression (LM), Principal Component Regression (PCR), Random Forest (RF) and Support Vector Machine (SVM), with nested cross-validation were implemented to capture the relationship between log-transformed CRP and variables of interest. PCR is a linear regression model which uses principal components as regressors instead of explanatory variables. RF is an ensemble learning model, where each base learner is a decision tree that is trained by Classification And Regression Tree (CART) algorithm. Only a subset of features is randomly selected and considered when splitting nodes; the final prediction of RF is the average over predictions from all trees in the model. SVM is a non-linear regression method that fits a linear hyperplane in the original or feature space based on the support vectors. *R*^2^ was used to evaluate the model performance, where bar plots and error bars represent means and standard deviations of *R*^2^ values for the training set, respectively, and red dots represent mean *R*^2^ values for the test set. The RF shows best performance among the four models for **A)** all subjects, **B)** female, and **C)** male.

**Figure S15. Variable Importance with PHQ-9 and Association with CRP**

**
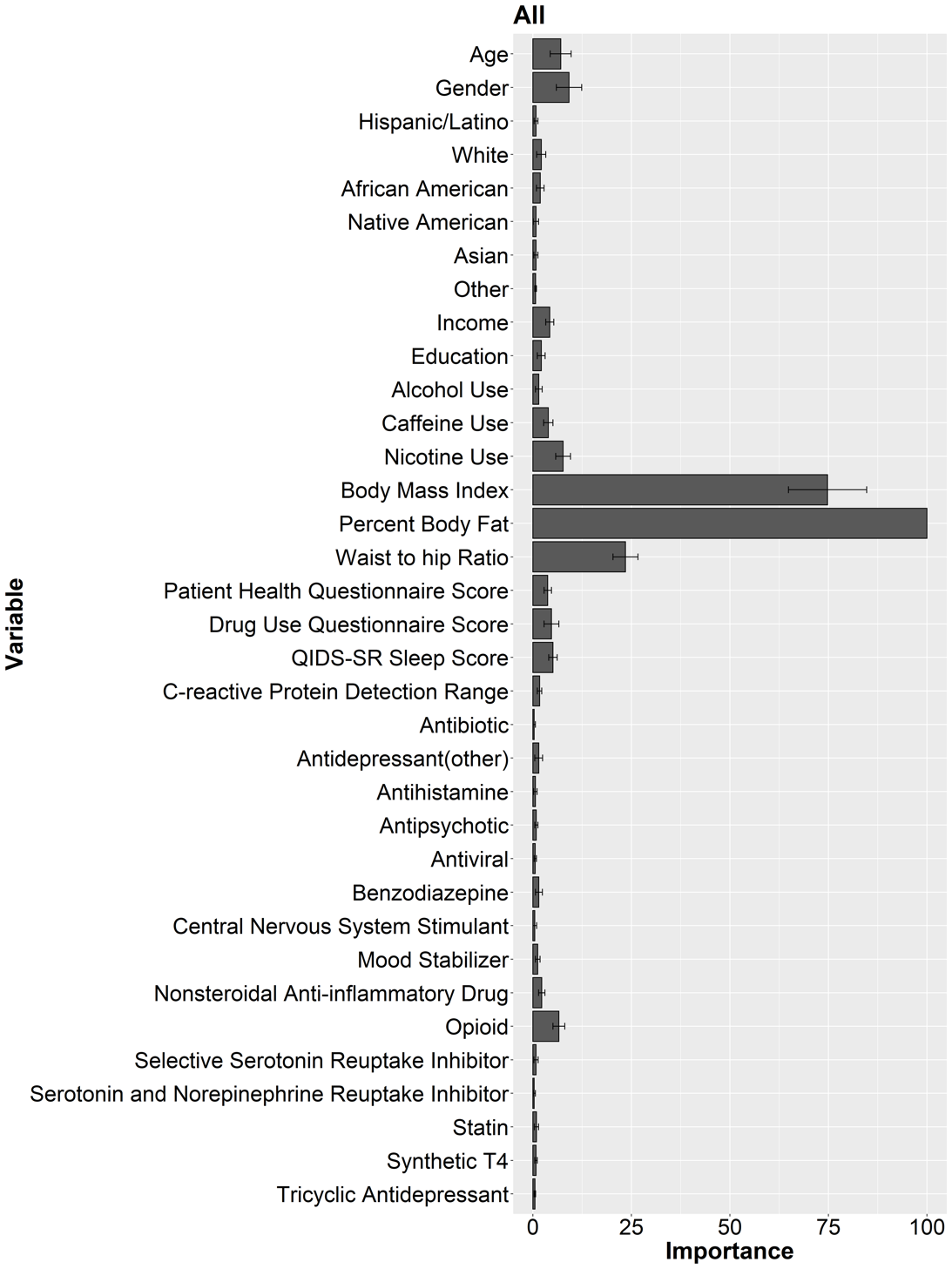
**

**Figure S15A.** Variable Importance for All dataset.


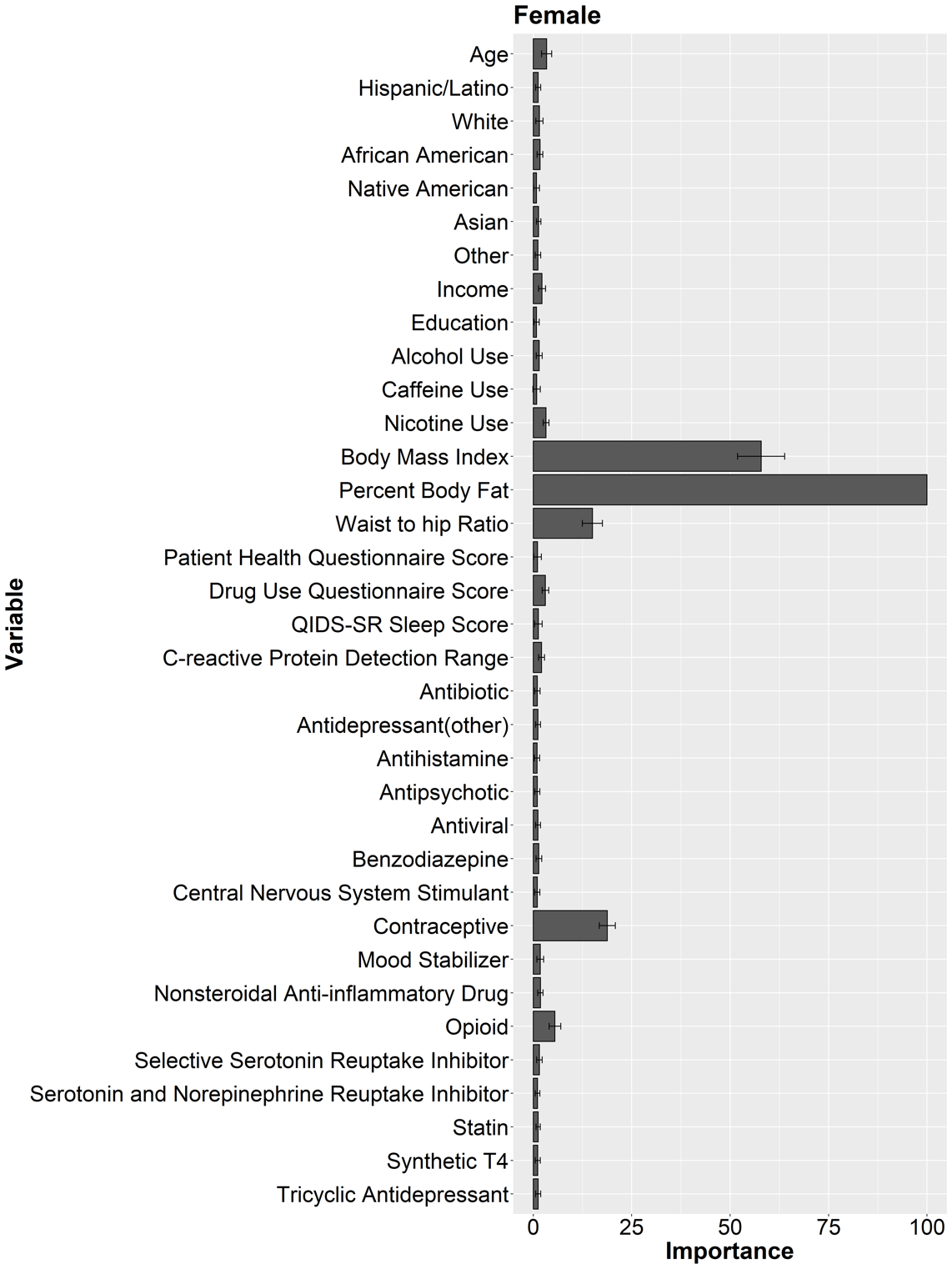


**Figure S15B.** Variable Importance for Female dataset.

**
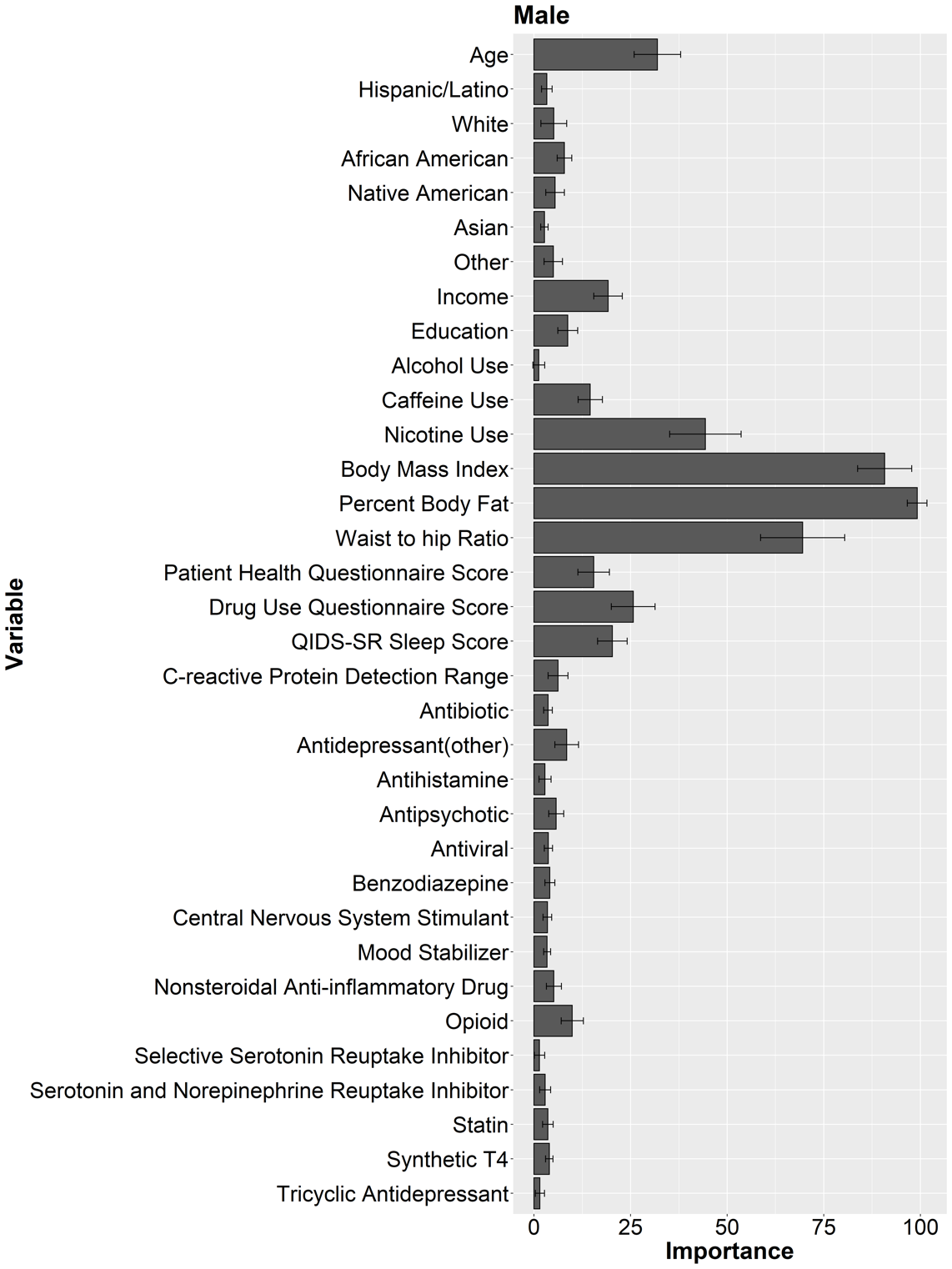
**

**Figure S15C.** Variable Importance for Male dataset.

**Figure S15. Variable Importance by Random Forest.** Variable importance on CRP concentrations was estimated by RF in **A)** all subjects, **B)** female, and **C)** male. To determine the importance of factors that were associated with CRP, we estimated variable importance by RF. The obesity indices, PBF and BMI, had the strongest association with CRP concentrations in both females and males. Use of OC and opioid medications were also identified as relatively important variables.

**Figure S16. Partial Dependent Plot for Body Mass Index (BMI) with PHQ9**

(A) All Subjects (B) Female Subjects (C) Male Subjects

**
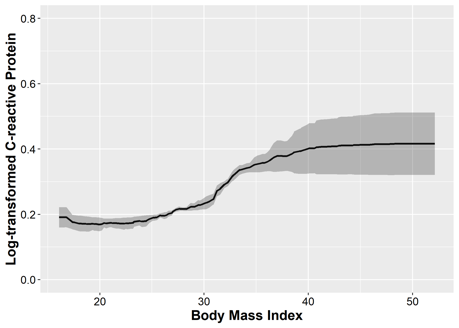

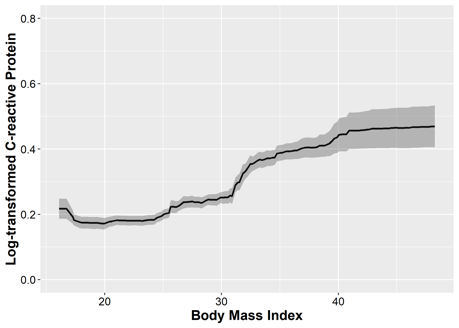

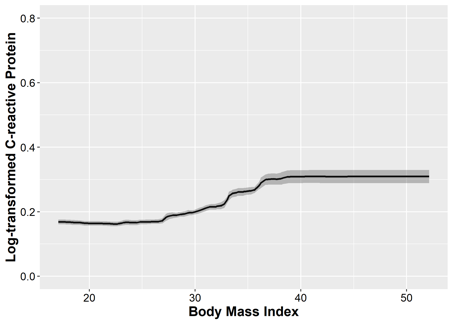
**

**Figure S16. Partial Dependent Plot- Body Mass Index (BMI). A)** This plot shows the non-linear relationship between BMI and CRP. CRP significantly increases with BMI in both **B)** females and **C)** males. To reveal the marginal effect of BMI on CRP, RF was used to generate a partial dependence plot. CRP increases in a non-linear fashion when BMI is higher than 30 in females but shows a relatively linear increase for males.

**Figure S17. Partial Dependent Plot for Percent Body Fat (PBF) with PHQ9**

(A) All Subjects (B) Female Subjects (C) Male Subjects

**
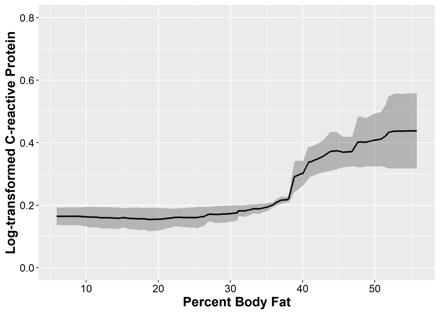

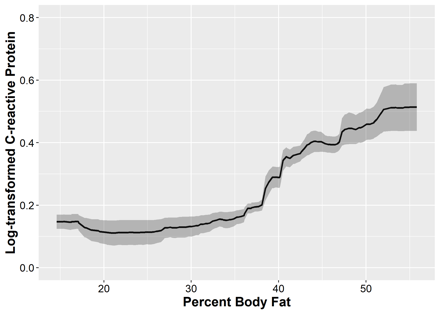

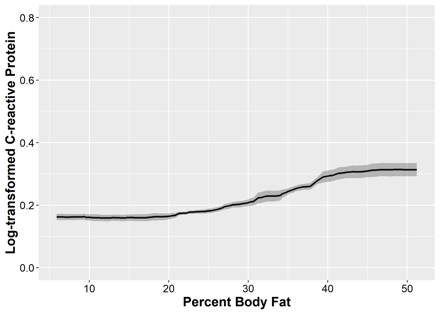
**

**Figure S17. Partial Dependent Plot- Percent Body Fat (PBF). A)** This plot shows the non-linear relationship between PBF and CRP. CRP significantly increases with PBF in both **B)** females and **C)** males. To reveal the marginal effect of PBF on CRP, RF was used to generate a partial dependence plot. CRP increases in a non-linear fashion when percent body fat is higher than 40 in females but shows a relatively linear increase for males.

**Figure S18. Partial Dependent Plot for Waist-to-Hip Ratio (WHR) with PHQ9**

(A) All Subjects (B) Female Subjects (C) Male Subject

**
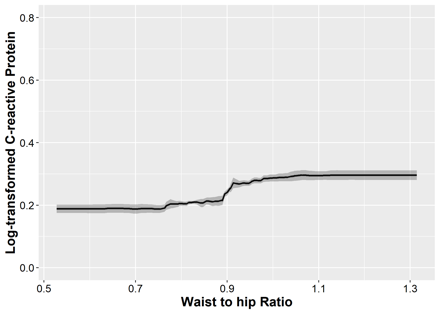

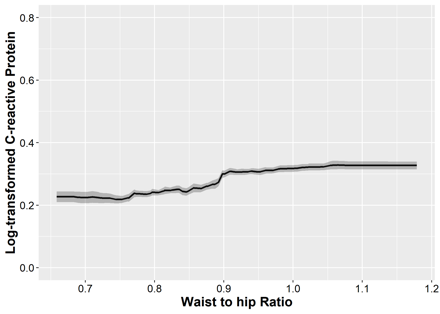

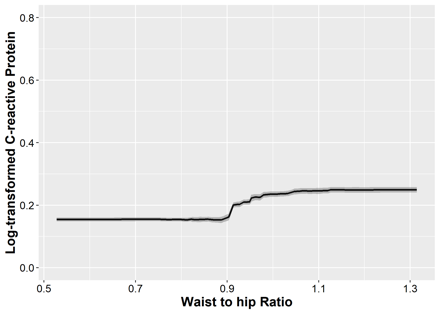
**

**Figure S18. Partial Dependent Plot- Waist-to-Hip ratio. A)** This plot shows the non-linear relationship between WHR and CRP. CRP increases with WHR increase. Both **B)** females and **C)** males only show relatively slight increases.

**Figure S19. Obesity Index Distribution**

1. **B) C)**

**
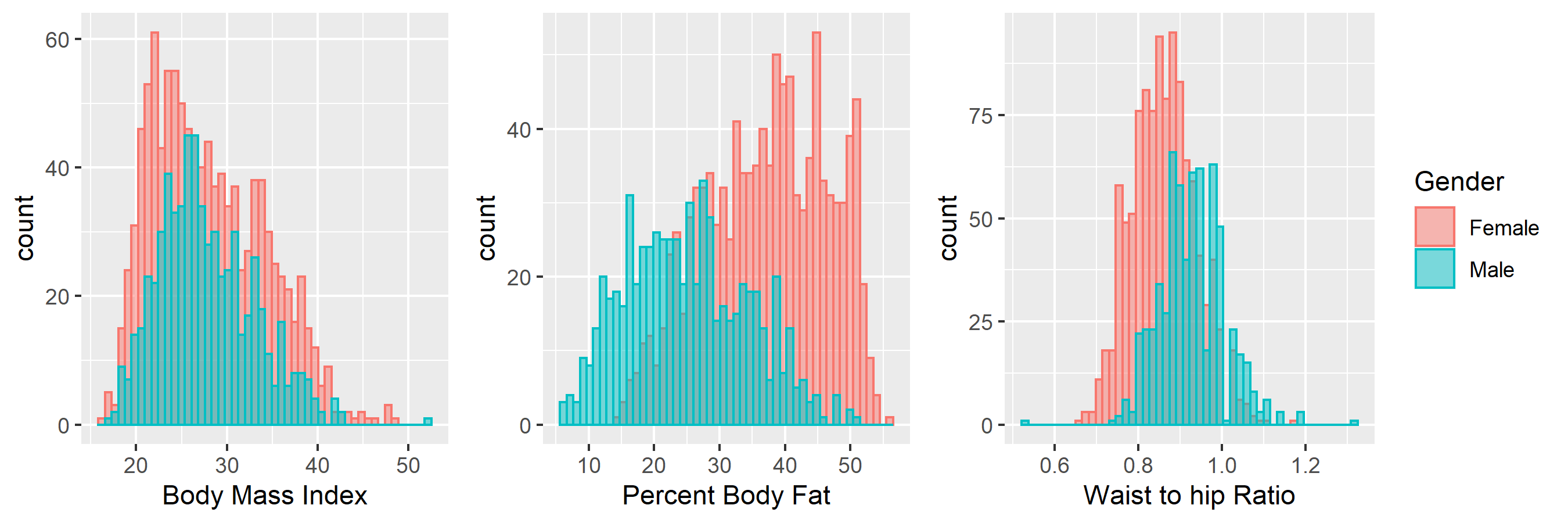
**

**Figure S19. Distribution of Obesity Indices in Females and Males.** Secondary analysis of female and male subjects for distribution of obesity indices, BMI, PBF and WHR. The distributions of BMI for males and females were similar, and females had higher PBF, on average. while WHR was greater in males. **A)** BMI distribution for both females and males. **B)** PBF distribution for both females and males. **C)** WHR ratio distribution for both females and males**.**

**Figure S20. Correlation between PHQ and OASIS**


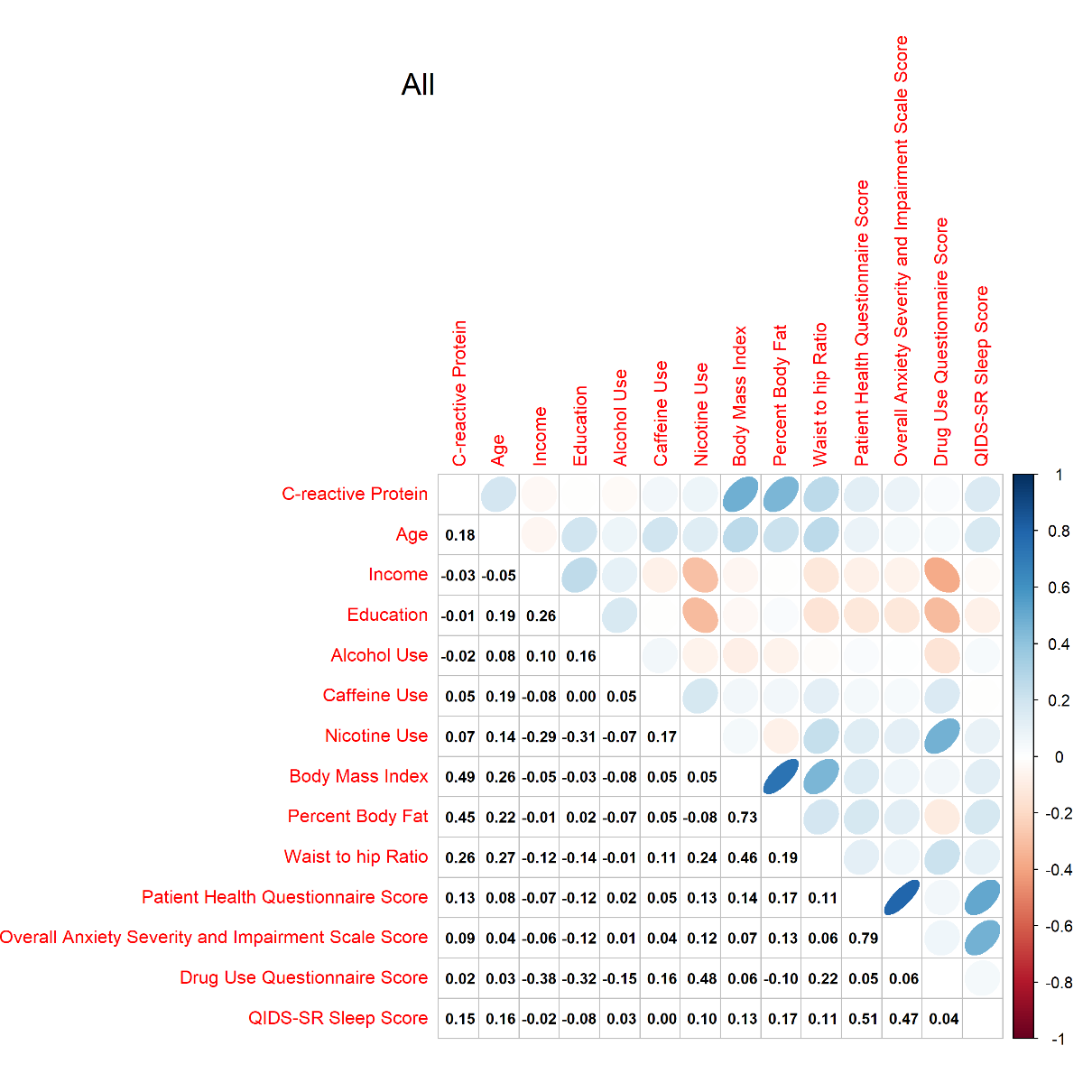


**Figure S20A.** Correlation plot between OASIS and other variables for All dataset.


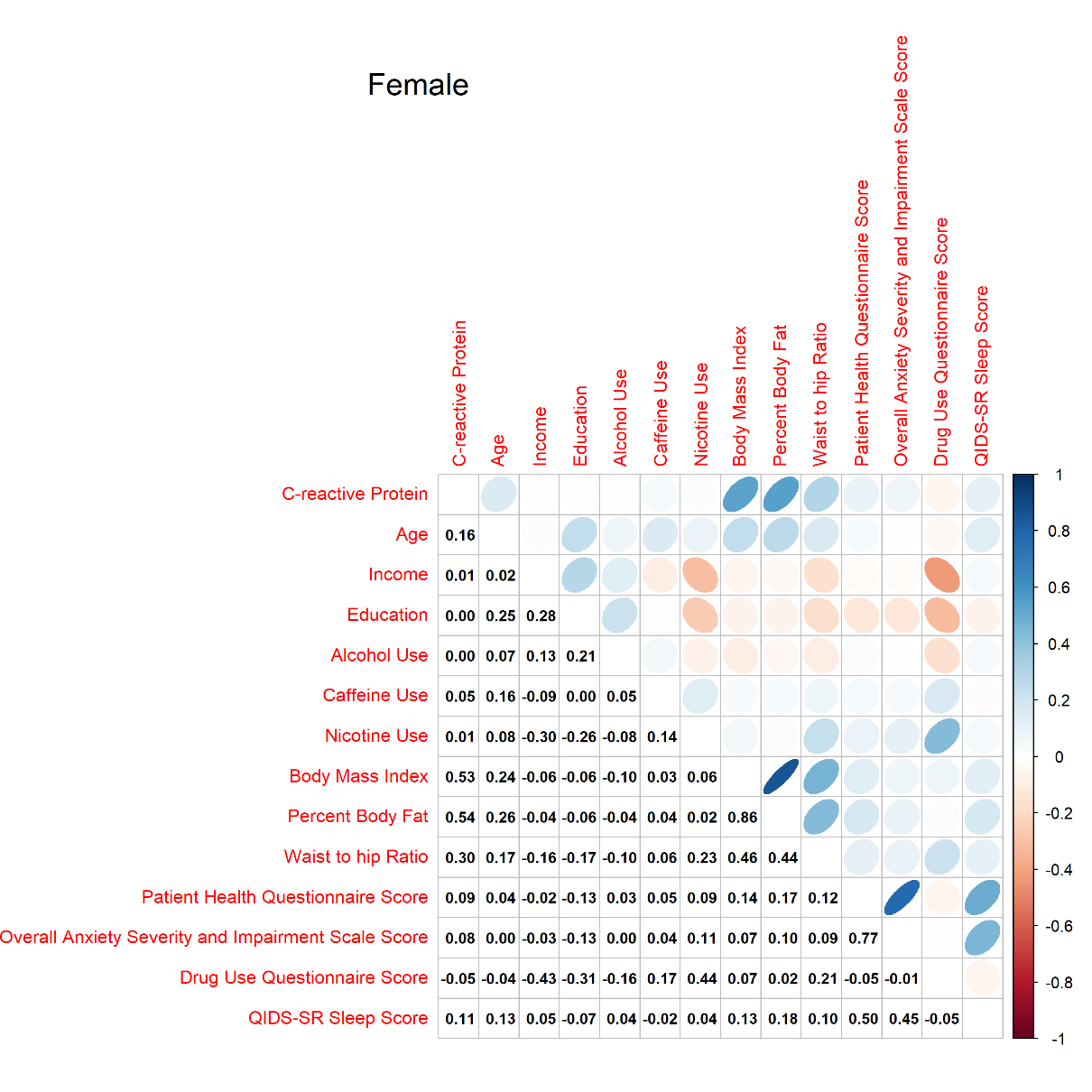


**Figure S20B.** Correlation plot between OASIS and other variables for Female dataset.


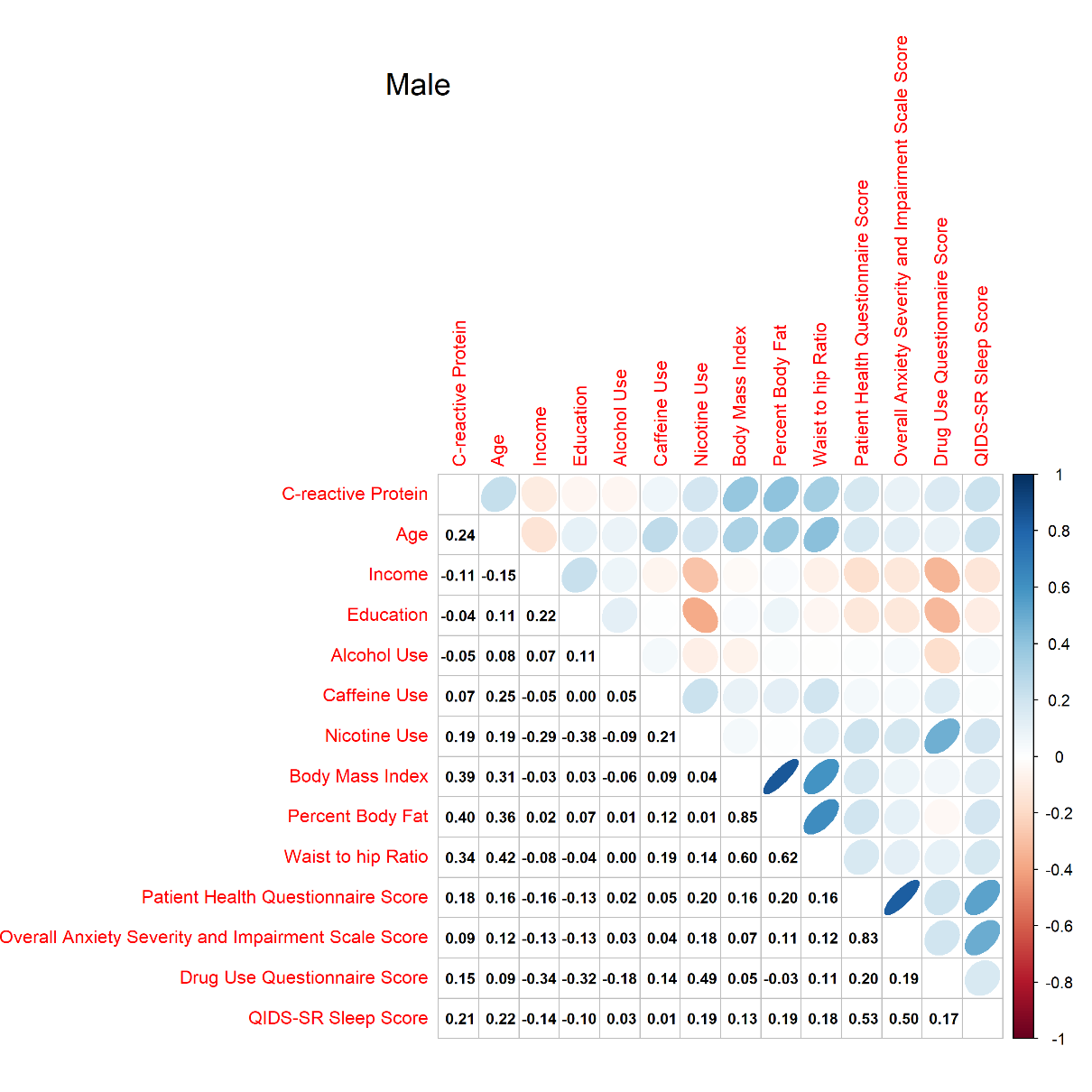


**Figure S20C.** Correlation plot between OASIS and other variables for Male dataset.

**Figure S20. Correlation Plots for OASIS.** CRP was not significantly correlated with anxiety as based on the Overall Anxiety Severity and Impairment Scale (OASIS). When OASIS was analyzed together with PHQ-9, the results were similar to those obtained with PHQ-9. OASIS and PHQ-9 were highly correlated for All (0.79), Female (0.77), and Male (0.83).
